# Lipogenesis promotes mitochondrial fusion and maintains cancer stemness in human NSCLC

**DOI:** 10.1172/jci.insight.158429

**Published:** 2023-03-22

**Authors:** Zhen Liu, Jiaxin Lei, Tong Wu, Weijie Hu, Ming Zheng, Ying Wang, Jingdong Song, Hang Ruan, Lin Xu, Tao Ren, Wei Xu, Zhenke Wen

**Affiliations:** 1 Jiangsu Key Laboratory of Infection and Immunity, Institutes of Biology and Medical Sciences, Soochow University, Suzhou, Jiangsu, China.; 2 State Key Laboratory of Infectious Disease Prevention and Control, National Institute for Viral Disease Control and Prevention, Chinese Center for Disease Control and Prevention, Beijing, China.; 3 Department of Immunology, Zunyi Medical University, Zunyi, Guizhou, China.; 4 Special Key Laboratory of Gene Detection & Therapy of Guizhou Province, Zunyi, Guizhou, China.; 5 Department of Respiratory Medicine, Shanghai Sixth People’s Hospital Affiliated to Shanghai Jiao Tong University School of Medicine, Shanghai, China.

**Keywords:** Oncology, Lung cancer, Mitochondria

## Abstract

Cancer stem-like cells (CSCs) are critically involved in cancer metastasis and chemoresistance, acting as one major obstacle in clinical practice. While accumulating studies have implicated the metabolic reprogramming of CSCs, mitochondrial dynamics in such cells remain poorly understood. Here we pinpointed OPA1^hi^ with mitochondrial fusion as a metabolic feature of human lung CSCs, licensing their stem-like properties. Specifically, human lung CSCs exerted enhanced lipogenesis, inducing OPA1 expression via transcription factor SAM Pointed Domain containing ETS transcription Factor (SPDEF). In consequence, OPA1^hi^ promoted mitochondrial fusion and stemness of CSCs. Such lipogenesis^hi^, SPDEF^hi^, and OPA1^hi^ metabolic adaptions were verified with primary CSCs from lung cancer patients. Accordingly, blocking lipogenesis and mitochondrial fusion efficiently impeded CSC expansion and growth of organoids derived from patients with lung cancer. Together, lipogenesis regulates mitochondrial dynamics via OPA1 for controlling CSCs in human lung cancer.

## Introduction

Lung cancer is the most common cause of cancer-related deaths worldwide (1, 2), with non–small cell lung cancer (NSCLC) as the primary type of clinical cases. Despite intensive studies on NSCLC pathogenesis and therapeutics, the prognosis of patients with NSCLC is still poor (3, 4). One critical reason is that many patients progress to metastatic lung cancer, for which there are few treatment options (5), leaving chemotherapy as a first-line choice. However, chemoresistance seems inevitable and remains a major hurdle in clinical practice (6–8). In recent decades, the link between cancer stem-like cells (CSCs) and metastatic lung cancer has been firmly established (9). Of note, accumulating evidence has demonstrated that chemotherapy does not eradicate CSCs but instead populates the CSC pool in patents (10). Thus, CSCs not only contribute to cancer metastasis, but also account for tumor chemoresistance and recurrence (11–13). However, precise mechanisms underpinning the stem-like properties of CSCs, which are relevant for cancer management, remain obscure.

CSCs are a small subset of cancer cells with self-renewal and tumor initiation capacities (14, 15). Tons of studies have identified stem-related molecules, including CD133, ALDH1, and OCT4, for CSCs (16–19). However, recent evidence has focused on the metabolic features of CSCs, which are essentially distinctive from non-CSC tumor cells (20). Aberrant glycolysis is well acknowledged as the metabolic characteristic for non-CSC tumor cells, also known as the Warburg effect, resulting in rapid ATP generation to support biosynthetic demands for outgrowth and metastasis (21, 22). Meanwhile, several studies have implicated an essential role of mitochondrial oxidative phosphorylation for CSC maintenance. Therefore, MYC and MCL1 cooperatively promote the expansion of breast cancer stem cells via mitochondria oxidative phosphorylation (20, 23, 24). CSCs within SCLC cell lines exert preferential use of oxidative phosphorylation over glycolysis to meet their energy demand (25). Our recent study identifies Notch1-enhanced mitochondrial oxidative phosphorylation in NSCLC CSCs (26). However, characteristics and mechanisms for aberrant mitochondrial metabolism of NSCLC CSCs remain largely undefined.

The crucial function of mitochondrial oxidative phosphorylation for CSCs reflects an optimal intracellular mitochondrial mass and quality, which are controlled by mitochondrial dynamics, a process that typically consists of mitochondrial fusion, fission, and subsequent degradation (27, 28). Indeed, mitochondrial morphology varies tremendously and changes rapidly via ‘‘mitochondria-shaping’’ proteins that regulate fusion and fission in response to metabolic cues (29). Accumulating studies have reported high levels of mitochondrial fission activity in some cancer cells and stem cells, associating with increased proliferation and invasiveness of cancer cells and with self-renewal and resistance to differentiation of stem cells (30). However, mitochondrial fission typically leads to subsequent degradation, which minimizes mitochondrial mass by eradicating dysfunctional mitochondria (27). In human NSCLC CSCs, mitophagy — a process for degrading dysfunctional mitochondria — is robust, resulting in the accumulation of mitochondrial DNA in lysosomes and subsequent activation of the TLR9-dependent Notch1 pathway (26). However, high mitophagy would eventually be conflicted with the high demand for mitochondrial oxidative phosphorylation for CSCs. Thus, an unknown mechanism should instruct elevated mitochondrial oxidative phosphorylation in CSCs.

In contrast to mitochondrial fission and mitophagy, mitochondrial fusion results in high mitochondrial metabolism for energy generation and biogenesis (31–33), making it reasonably relevant for mitochondrial oxidative phosphorylation. Mechanistically, mitochondrial fusion is mediated by 2 outer membrane (OM) proteins, including mitofusin 1 (MFN1) and MFN2, while optic atrophy 1 (OPA1) is the only dynamin-related protein (DRP) identified in the inner membrane (IM) (25, 34). Work by Bonnay et al. has demonstrated that metabolic reprogramming induced by mitochondrial fusion can be rate limiting for immortalizing tumor-initiating cells (TICs) of *Drosophila* brain tumors, triggering their irreversible dedication to tumorigenesis (35). However, the integral feature of mitochondrial fusion activity in human NSCLC CSCs is unclear.

In this study, we explored the mitochondrial fusion activity of NSCLC CSCs and pinpointed OPA1 as the gatekeeper for the stem-like properties of CSCs. Specifically, aberrant lipogenesis in CSCs induced high OPA1, instructing mitochondrial fusion activity and stem-like functions. Using the transcription factor–predicting database in combination with transcriptomic profiling, we identified SAM Pointed Domain containing ETS transcription Factor (SPDEF) as the critical transcription factor that induced the transcription of OPA1. Accordingly, blockade of lipogenesis and SPDEF inhibited the transcription of OPA1, resulting in minimal mitochondrial fusion activity and impaired stem properties of CSCs. Collectively, OPA1 bridges lipogenesis and mitochondrial dynamics in CSCs, promoting mitochondrial fusion activity and stem-like properties of human lung cancer.

## Results

### High mitochondrial fusion activity licenses stem-like properties of CSCs.

**Figure 1:**
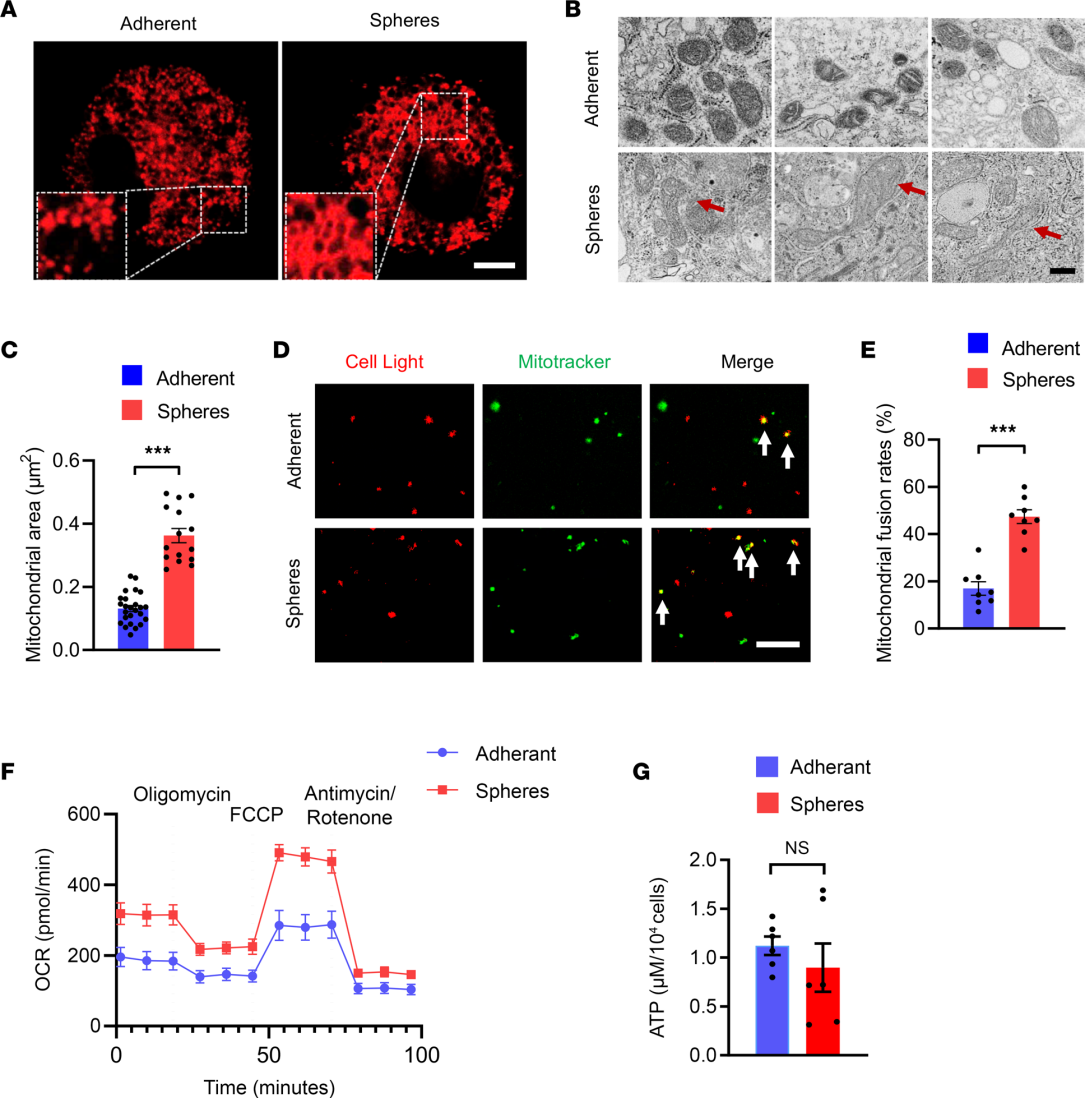
Higher mitochondrial fusion in CSCs. (**A**) Tumor spheres and adherent cells from A549 were stained with MitoTracker-Red for 15 minutes and detected for mitochondrial dynamics using confocal microscopy. Representative images from 5 independent experiments. Scale bar: 10 μm. (**B** and **C**) Tumor spheres and adherent cells from A549 were analyzed for mitochondrial ultrastructural by TEM and quantified for corresponding mitochondrial areas (μm^2^). Representative and mean ± SEM from 5 independent experiments. Scale bar: 500 nm. (**D** and **E**) Mitochondria from A549 tumor spheres or adherent cells were stained with CellLight-Red and MtioTracker-Green at a 1:1 ratio and detected for fusion activity. Representative and mean ± SEM from 8 independent experiments. Scale bar: 5 μm. (**F**) Oxygen consumption rate (OCR) of A549 tumor sphere and adherent cells. Mean ± SEM from 5 independent experiments. (**G**) Intracellular ATP levels in A549 tumor sphere and adherent cells. Mean ± SEM from 6 independent experiments. ***P < 0.001 with paired *t* test. Red arrows point to tubulated and elongated mitochondria. White arrows indicate the mitochondria where fusion occurs.

General cultures of human cancer cell lines retain a certain degree of heterogeneity (36). Specifically, tumor spheres cultured in nonadherent conditions with the stem cell–selective medium were used as CSCs, and the adherent cells were used as non-CSC tumor cells (37). We verified that tumor sphere cultures from A549 cells were enriched with stem-like cells by assessing the expression of stem-related genes, including OCT4, ALDH1, SOX2, and CD133 (Supplemental Figure 1; supplemental material available online with this article; https://doi.org/10.1172/jci.insight.158429DS1). To access the mitochondrial fusion activity in CSCs and non-CSC tumor cells, tumor spheres and adherent cells from A549 cells were stained with MitoTracker and imaged using confocal microscopy for mitochondrial morphology. While adherent cells contained mostly smaller rounded mitochondria, tumor spheres exerted tubulated and elongated ones (Figure 1A), indicating a higher mitochondrial fusion activity in NSCLC CSCs. We then conducted transmission electron microscopy (TEM) to examine mitochondrial ultrastructural differences in tumor spheres and adherent cells. Again, tubulated and elongated mitochondria with the increased mitochondrial area were consistently observed in tumor spheres, demonstrating higher mitochondrial fusion activity (Figure 1, B and C). To reassure such phenomena, the mitochondrial fusion rate was detected by in vitro mitochondrial fusion assay (38). The mitochondrial fusion rates were significantly higher with mitochondria from tumor spheres (Figure 1, D and E). Accordingly, mitochondrial oxygen consumption rates (OCR) were also higher in tumor spheres (Figure 1F), supporting an essential role of mitochondrial oxidative phosphorylation in CSC stemness. However, intracellular ATP levels were generally comparable between tumor spheres and adherent cells (Figure 1G), indicating a preferential use of mitochondrial oxidative phosphorylation for CSCs to generate ATP and glycolysis for non-CSCs to meet the energy demand.

**Figure 2:**
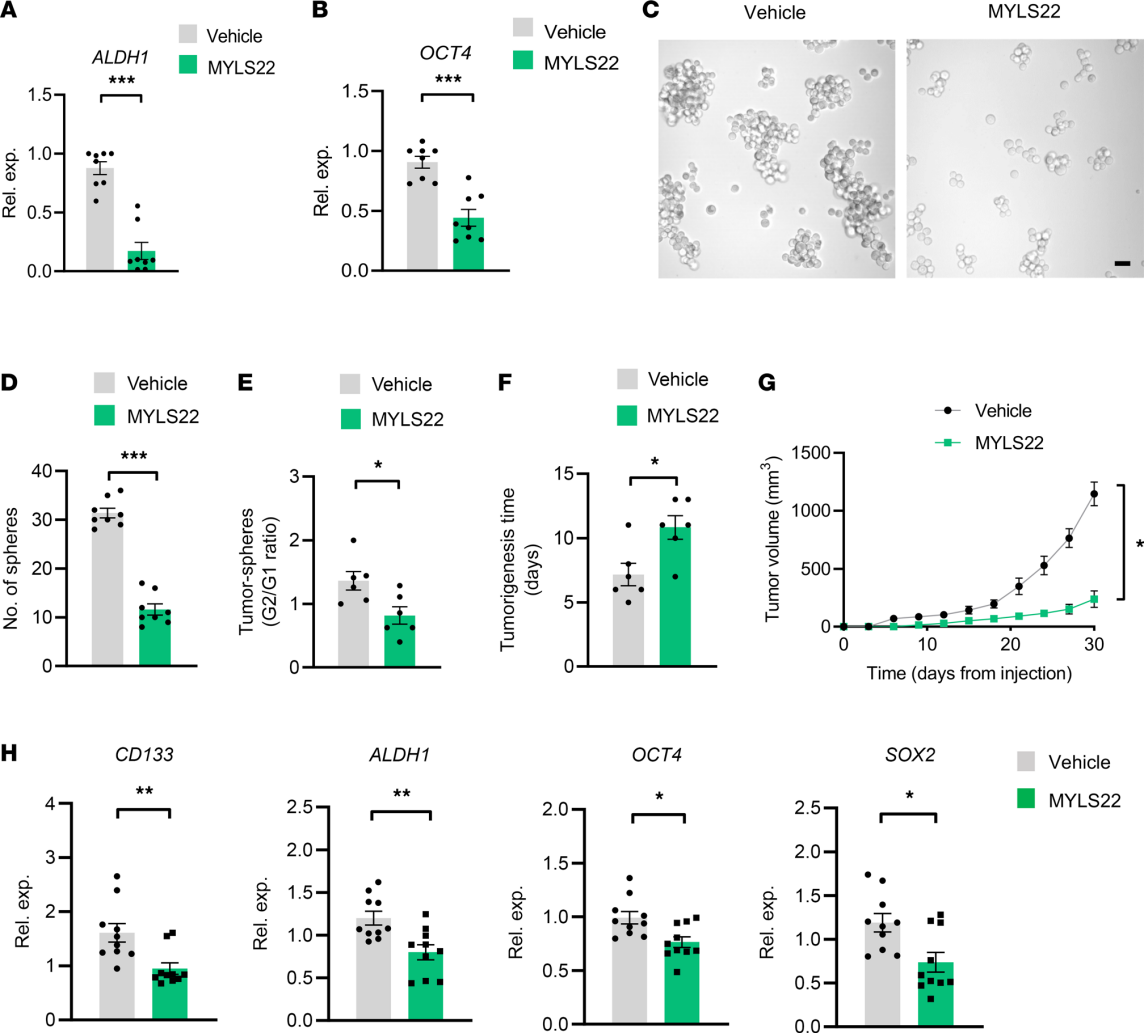
Mitochondrial fusion is critical for the stemness of CSCs. (**A** and **B**) A549 tumor spheres were treated with or without MYLS22 (40 μM) for 12 hours and detected for ALDH1 and OCT4 mRNA expressions by qPCR. Mean ± SEM from 8 independent experiments. (**C** and **D**) A549 tumor spheres with or without MYLS22 (40 μM) were detected for sphere formations. Representative and mean ± SEM from 8 independent experiments. Scale bar: 10 μm. (**E**) Ration of A549 tumor sphere formation at first (G1) and second (G2) generation after MYLS22 (40 μM) treatment. Mean ± SEM from 8 independent experiments. (**F** and **G**) A549 tumor spheres with or without MYLS22 (40 μM) were tested for tumor initiation and growth in NSG mice. Mean ± SEM from 6 mice in each group. (**H**) Treatment with MYLS22 (40 μM) reduced expressions of stem-related genes in A549 CSC tumors. Mean ± SEM from 10 tumors in each group. *P < 0.05, **P < 0.01, ***P < 0.001 with paired *t* test.

To detect the functional consequence of high mitochondrial fusion activity in CSCs, tumor spheres were treated with MYLS22, a first-in-class inhibitor for mitochondrial fusion (39), and analyzed for sphere formation, self-renewal, and tumor initiation. MYLS22 treatment efficiently reduced expression levels of ALDH1 and OCT4 (Figure 2, A and B) and inhibited sphere formations (Figure 2, C and D). Of note, MYLS22 abrogated sphere formation of the second generation of tumor spheres (Figure 2E), indicating a persistent impairment of stem-like properties. Finally, MYLS22 significantly delayed the tumor initiation and inhibited tumor growth of CSCs in NSG mice (Figure 2, F and G). Of note, MYLS22 treatment efficiently reduced the expression levels of stem-related genes in CSC tumors (Figure 2H), suggesting that the MYLS22-mediated inhibition of tumor growth was due to decreased stem-like cells. Together, NSCLC CSCs exert high mitochondrial fusion activity, licensing their stem-like properties.

### High OPA1 confers high mitochondrial fusion activity of CSCs.

**Figure 3:**
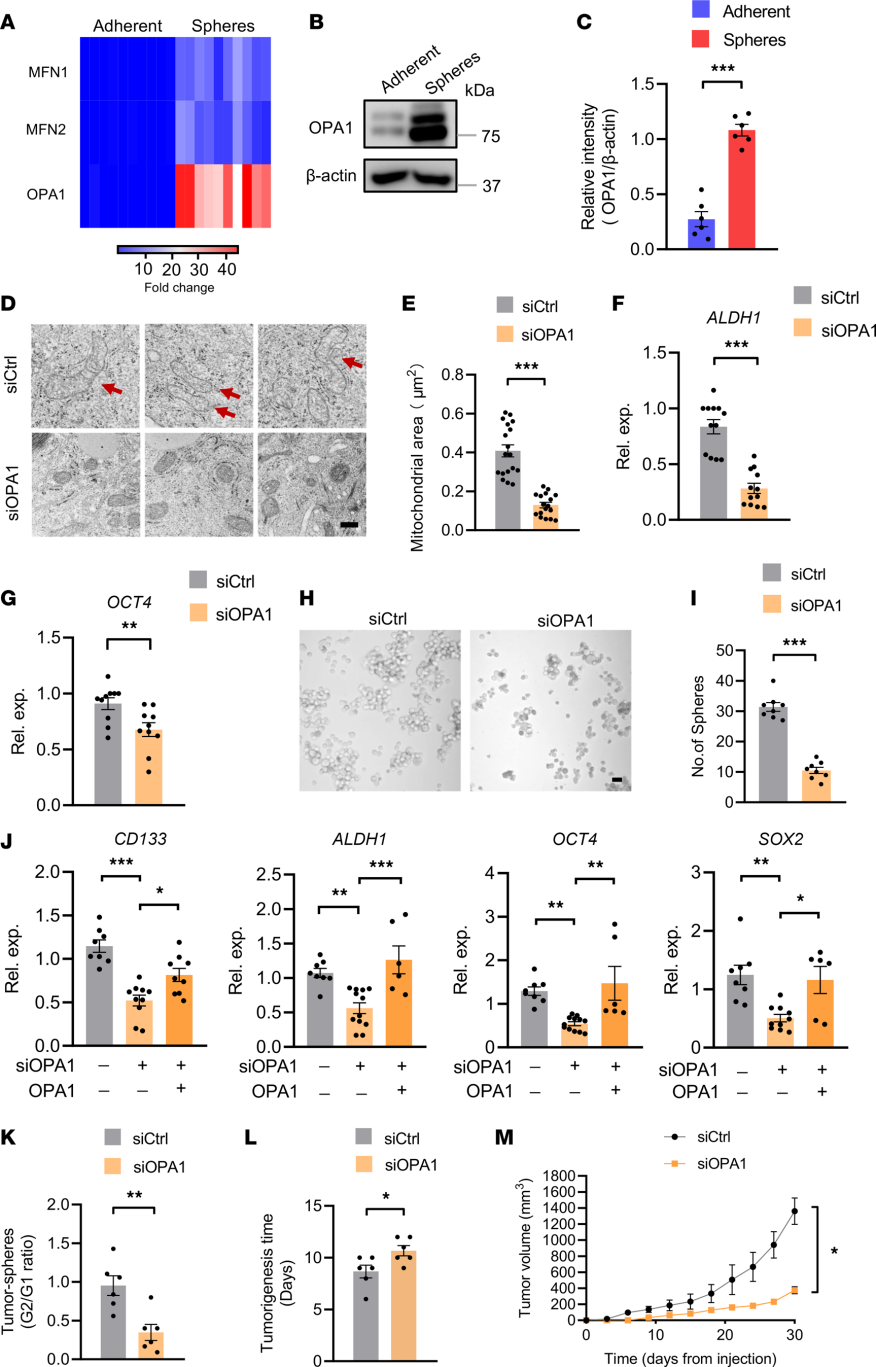
OPA1^hi^ licenses mitochondrial fusion for CSCs stem properties. (**A**) Heatmap showing MFN1, MFN2, and OPA1 mRNA expressions in A549 tumor spheres and adherent cells. Data from 10 independent experiments. (**B** and **C**) Immunoblot analysis of OPA1 protein in A549 tumor spheres and adherent cells. Representative and mean ± SEM from 5 independent experiments. (**D** and **E**) A549 tumor spheres with or without OPA1 genetic knockdown were detected for mitochondrial ultrastructure with TEM and analyzed for mitochondrial areas. Representative and mean ± SEM from 5 independent experiments. Scale bars: 500 nm. Red arrows point to tubulated and elongated mitochondria. (**F** and **G**) A549 tumor spheres were transfected with OPA1 siRNA or the control for 48 hours and detected for ALDH1 and OCT4 mRNA expression by qPCR. Mean ± SEM from 10–12 independent experiments. (**H** and **I**) A549 tumor spheres with or without OPA1 genetic knockdown were analyzed for sphere formations. Representative and mean ± SEM from 8 independent experiments. (**J**) A549 tumor spheres were transfected with a second OPA1 siRNA in the presence or absence of the reintroduction of OPA1 overexpression, and they were detected for expression of the indicated stem-related genes. Mean ± SEM from 6–10 independent experiments. (**K**) Genetic knockdown of OPA1 results in persistent damage with sphere formations of A549 tumor spheres. Mean ± SEM from 6 independent experiments. (**L** and **M**) A549 tumor spheres with or without OPA1 genetic knockdown were detected for tumor initiation and growth in NSG mice. Mean ± SEM from 6 independent experiments. *P < 0.05, **P < 0.01, ***P < 0.001 with paired *t* test (**C**–**I** and **K**–**M**) and ANOVA plus Tukey’s method (**J**).

To explore mechanisms underpinning the high mitochondrial fusion activity in CSCs, tumor spheres and adherent cells were monitored for expressions of fusion proteins, including MFN1, MFN2, and OPA1. Compared with MFN1 and MFN2, which were slightly elevated in tumor spheres, the transcriptome of OPA1 was dramatically increased in CSCs (Figure 3A). High OPA1 in CSCs was confirmed with immunoblot, showing approximately 5-fold higher relative intensity in tumor spheres (Figure 3, B and C).

To determine whether high OPA1 was critically involved in high mitochondrial fusion activity and stem-like properties of CSCs, tumor spheres were transfected with OPA1 siRNA or the control and monitored for mitochondrial fusion activity with TEM. Genetic knockdown of OPA1 using siRNA efficiently reduced OPA1 expressions (Supplemental Figure 2), impairing mitochondrial fusion in tumor spheres (Figure 3, D and E).

In line with the essential role of mitochondrial fusion for stem-like properties (Figure 1), knockdown of OPA1 inhibited expressions of stem-related gene ALDH1 and OCT4 in tumor spheres (Figure 3, F and G), accompanied by a substantial loss of sphere-formation capacity (Figure 3, H and I). These findings were confirmed with experiments using a second OPA1 siRNA and the reintroduction of OPA1 by overexpression, showing OPA1-licensed expressions of stem-related genes in tumor spheres (Figure 3J). Furthermore, OPA1 knockdown resulted in persistent impairment of stem-like properties of CSCs, as the sphere formation of the second generation was significantly inhibited (Figure 3K). OPA1-depleted tumor spheres also exerted delayed tumorigenicity and compromised outgrowth in NSG mice (Figure 3, L and M). Thus, OPA1 is substantially upregulated in CSCs, driving high mitochondrial fusion activity and stem-like properties.

### CSCs are metabolically high lipogenesis.

**Figure 4:**
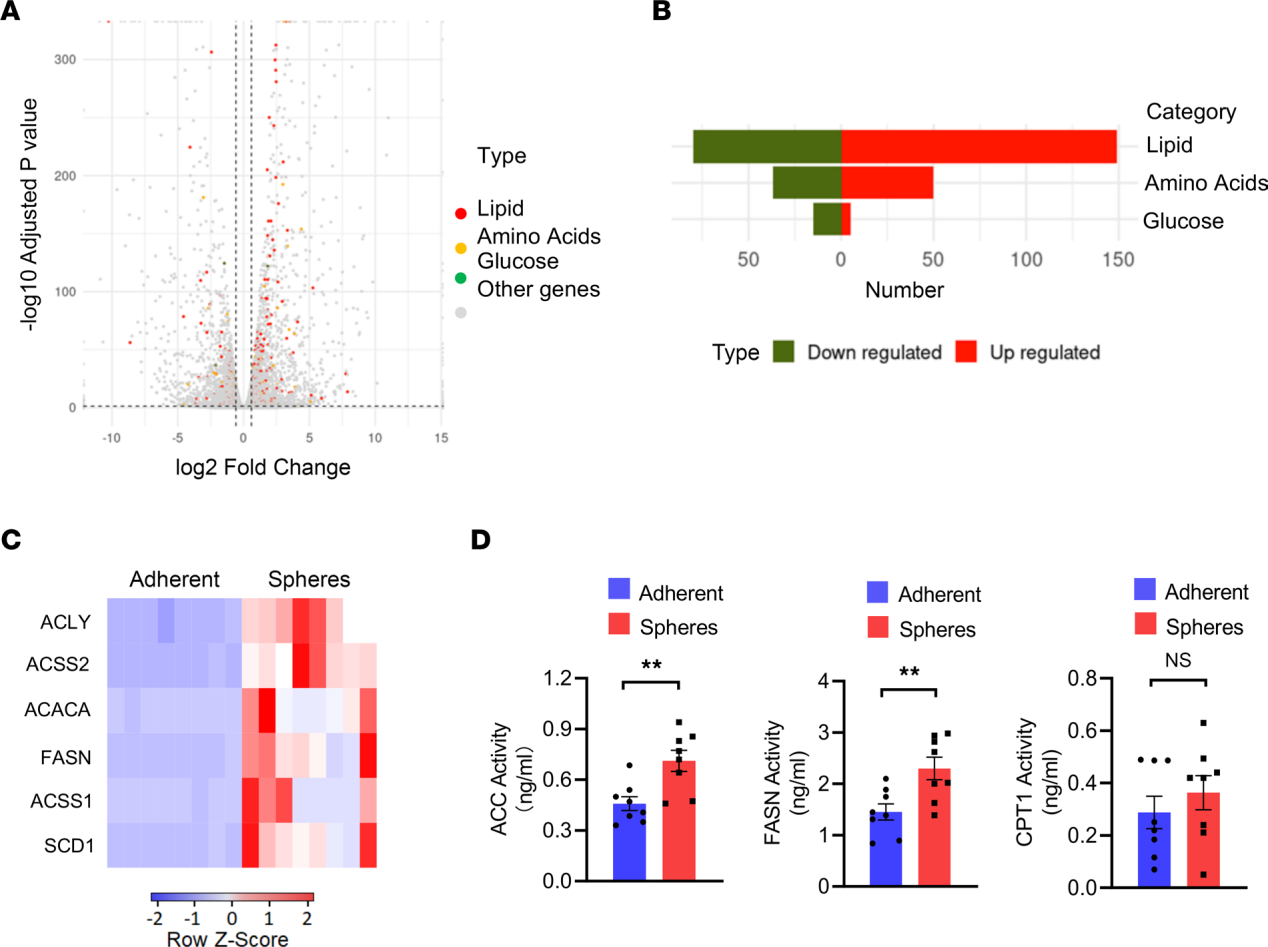
Elevated expression and activities of lipogenic enzymes in CSCs. (**A**) Volcano plot shows metabolic signatures by quantifying glucose (green), lipid (red), and amino acids (yellow) metabolic-related genes that are differentially expressed in A549 tumor spheres compared with adherent cells. RNA-Seq from 3 samples in each group. (**B**) Histogram showing the number of changed genes that are related to glucose, lipid, and amino acids metabolism in A549 tumor spheres and adherent cells. RNA-Seq from 3 samples in each group. (**C**) mRNA expressions of the indicated lipogenesis-related genes in A549 tumor spheres and adherent cells. Shown from 8 independent experiments. (**D**) The enzymic activities of ACC, FASN, and CPT1 in A549 tumor spheres and adherent cells. Mean ± SEM from 8 independent experiments. **P < 0.01 with paired *t* test.

As part of intracellular metabolism, mitochondrial fusion is tightly controlled by metabolic pathways (30, 40). To understand why OPA1 with high mitochondrial fusion was aberrant in CSCs, tumor spheres and adherent cells were tested for metabolic signatures using RNA-Seq. We found that tumor spheres were metabolically distinctive from adherent cells, showing the most dramatic changes with lipid metabolic–related genes (Figure 4, A and B). Of note, lipid metabolic–related genes with elevated expressions were dominantly involved in lipogenesis (Supplemental Table 1 and Supplemental Figure 3A). Quantitative PCR (qPCR) experiments confirmed that genes involved in lipogenesis, but not lipid β-oxidation, was fundamentally elevated in CSCs (Figure 4C and Supplemental Figure 3B). Furthermore, enhanced activities of lipogenic enzymes ACC and FASN, as well as a comparable activity of lipid catabolic CPT1, were observed in tumor spheres (Figure 4D), indicating an enhanced lipogenesis and an unchanged lipid catabolism in NSCLC CSCs.

**Figure 5:**
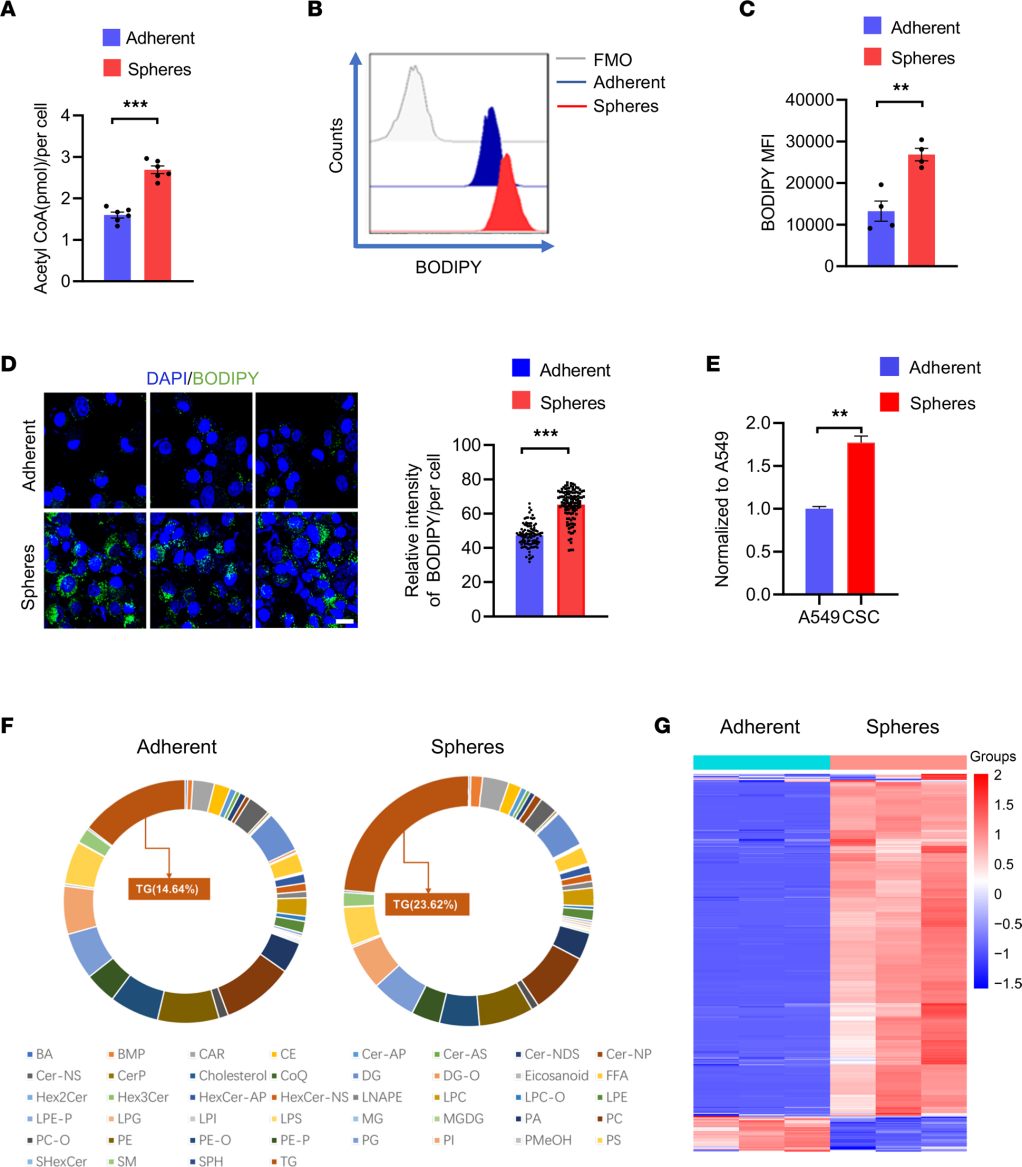
Aberrant lipogenic metabolism in CSCs. (**A**) Quantification of acetyl-CoA in A549 tumor spheres and adherent cells. Mean ± SEM from 6 independent experiments. (**B** and **C**) Intracellular lipid depositions were analyzed with BODIPY (493/503) staining in A549 tumor spheres and adherent cells. Representative and mean ± SEM from 4 independent experiments. (**D**) A549 tumor spheres and adherent cells were stained with BODIPY (493/503) and visualized with confocal microscopy. Representative and mean ± SEM from 5 independent experiments. Nuclei were stained with DAPI. Scale bar: 10 μm. (**E**) Metabolomic analysis showing increased lipid content in A549 tumor spheres. Metabolomic data from 3 samples in each group. (**F** and **G**) TG as the dominant lipid subtyping was apparently accumulated in A549 tumor spheres. Metabolomics from 3 samples in each group. **P < 0.01, ***P < 0.001 with paired *t* test.

To confirm the distinctive lipogenesis activity in tumor spheres and adherent cells, such cells were tested for intracellular acetyl-CoA and accumulation of lipid droplets. We found significantly higher levels of acetyl-CoA in tumor spheres (Figure 5A). Lipid droplets determined by BODIPY staining were also 2- to 3-fold higher in tumor spheres compared with adherent cells (Figure 5, B and C). Intracellular lipid droplets at a single-cell level were visualized using confocal microscopy, and stronger staining of BODIPY was observed in tumor spheres (Figure 5D). Finally, metabolomic analyses of lipid in tumor spheres and adherent cells were performed, confirming the increased load of lipid metabolites in tumor spheres (Figure 5E). Triglycerides (TG) as the dominant lipid metabolites were widely increased in tumor spheres (Figure 5, F and G). Thus, CSCs are lipogenesis^hi^, metabolically distinguishable from non-CSCs.

### Lipogenesis^hi^ licenses high OPA1 and stem-like properties of CSCs.

**Figure 6:**
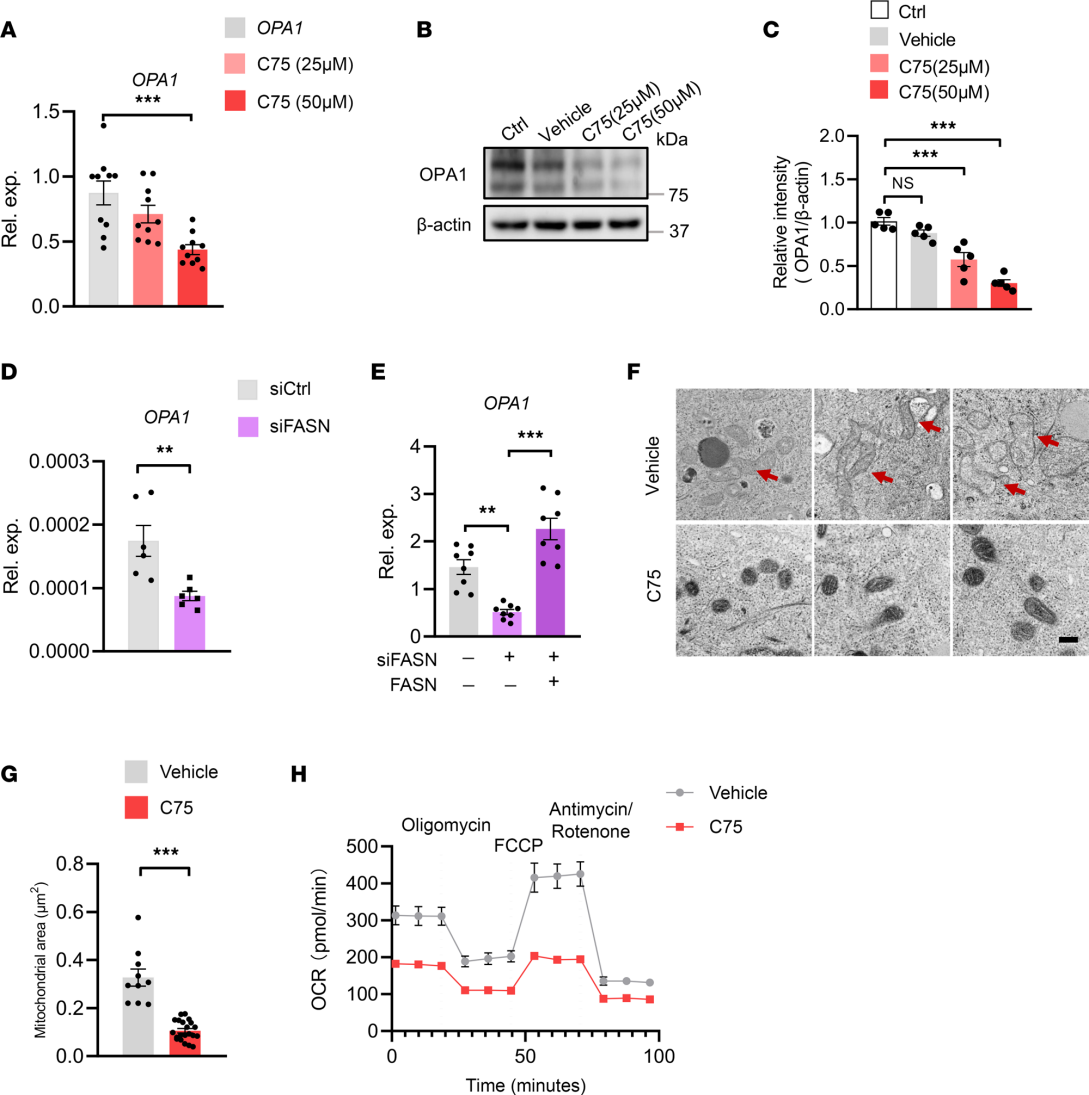
Lipogenesis drives OPA1 expression and mitochondrial fusion in CSCs. (**A**) A549 tumor spheres were treated with the indicated doses of C75 for 12 hours and detected for OPA1 expression by qPCR. Mean ± SEM from 10 independent experiments. (**B** and **C**) A549 tumor spheres were treated with an increasing dose of C75 for 24 hours and detected for OPA1 protein with immunoblots. Representative and mean ± SEM from 5 independent experiments. (**D**) A549 tumor spheres were transfected with FASN siRNA or the control and detected for OPA1 expressions by qPCR after 12 hours. Mean ± SEM from 6 independent experiments. (**E**) A549 tumor spheres were transfected using a second FASN siRNA with or without the reintroduction of FASN by overexpression and were analyzed for OPA1 expression. Mean ± SEM from 8 independent experiments. (**F** and **G**) A549 tumor spheres with or without C75 treatment (50 μM) were detected for mitochondria morphology and areas. Scale bars: 500 nm. Representative and mean ± SEM from 5 independent experiments. Red arrows point to tubulated and elongated mitochondria. (**H**) A549 tumor spheres with or without C75 treatment (50 μM) were detected for mitochondrial OCR using Seahorse assay. Mean ± SEM from 5 independent experiments. **P < 0.01, ***P < 0.001 with ANOVA plus Tukey’s method (**A**–**C** and **E**) and paired *t* test (**D** and **G**).

Given lipogenesis^hi^ as a metabolic signature of CSCs, we next determined whether lipogenesis^hi^ led to high OPA1 expression and mitochondrial fusion activity. Administration of C75, a selective inhibitor of lipogenesis enzyme FASN, reduced mRNA and protein expressions of OPA1 in a dose-dependent manner (Figure 6, A–C). In contrast, 2-DG as a glucose analog to block glucose metabolism, Etomoxir as a lipid β-oxidation inhibitor by selectively targeting CPT1, and BPTES as a glutamine metabolism inhibitor by selectively targeting glutaminase GLS1 exerted no significant effects on OPA1 expression in tumor spheres (Supplemental Figure 4A). The C75-mediated inhibition of OPA1 was selective, as C75 exerted no significant effect on the expression of MFN2 in tumor spheres (Supplemental Figure 4, B and C). Furthermore, FASN was genetically knocked down using siRNA in tumor spheres (Supplemental Figure 4D), and this essentially inhibited the transcriptome of OPA1, resulting in a 50% reduction of OPA1 mRNA levels (Figure 6D). Such findings were confirmed with experiments using a second FASN siRNA and the reintroduction of FASN by overexpression, showing the critical role of FASN in regulating OPA1 expressions in CSCs (Figure 6E). Accordingly, C75 treatment also abrogated the mitochondrial fusion in tumor spheres (Figure 6, F and G), together with an impaired mitochondrial OCR activity of such CSCs (Figure 6H).

**Figure 7:**
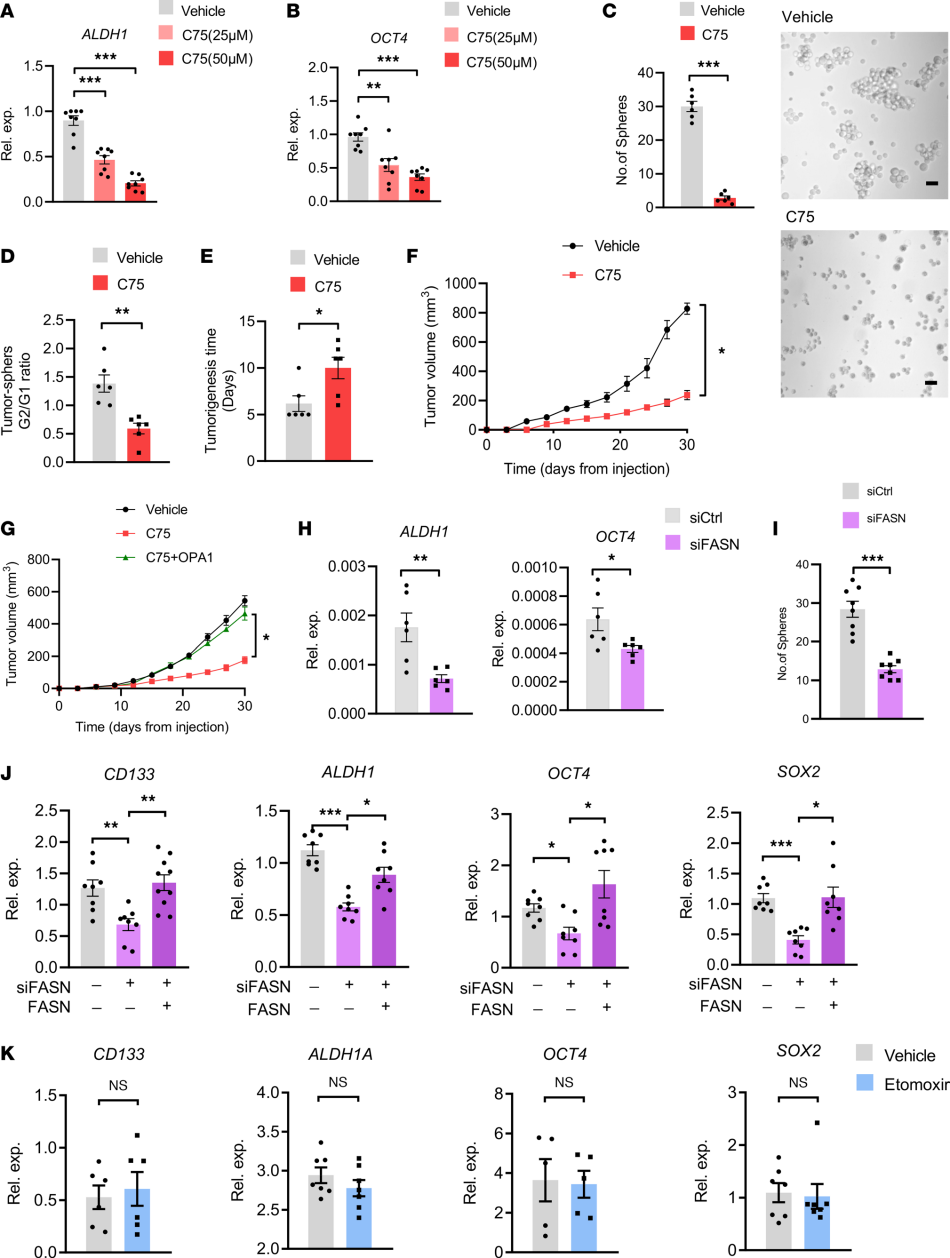
Lipogenesis controls the stem-like properties of CSCs. (**A** and **B**) A549 tumor spheres were treated with an increasing dose of C75 and detected for ALDH1 and OCT4 expressions by qPCR after 12 hours. Mean ± SEM from 8 independent experiments. (**C**) A549 tumor spheres with or without C75 treatment (50 μM) were analyzed for sphere formations. Representative and mean ± SEM from 5 independent experiments. Scale bar: 10 μm. (**D**) Ration of A549 tumor sphere formation for the first (G1) and second (G2) generations after C75 (50 μM) treatment. Mean ± SEM from 5 independent experiments. (**E** and **F**) A549 tumor spheres with or without C75 treatment (50 μM) were analyzed for tumor initiation and growth in NSG mice. Mean ± SEM from 6 mice in each group. (**G**) A549 tumor spheres with or without C75 treatment (50 μM) plus OPA1 overexpression were analyzed for tumor growth in NSG mice. Mean ± SEM from 6 mice in each group. (**H**) A549 tumor spheres with or without FASN knockdown were detected for ALDH1 and OCT4 mRNA by qPCR. Mean ± SEM from 6 independent experiments. (**I**) Knockdown of FASN in A549 tumor spheres inhibited sphere-formations. Mean ± SEM from 8 independent experiments. (**J**) A549 tumor spheres were transfected using a second FASN siRNA with or without the reintroduction of FASN and were analyzed for expression of the stem-related genes. Mean ± SEM from 8 independent experiments. (**K**) A549 tumor spheres were treated with or without CPT1 inhibitor Etomoxir (100 μM) and analyzed for expression of the stem-related genes. Mean ± SEM from 5–7 independent experiments. *P < 0.05, **P < 0.01, ***P < 0.001 with ANOVA plus Tukey’s method (**A**, **B**, and **J**) and paired *t* test (**C**–**I** and **K**).

In line with the crucial function of mitochondrial fusion in stem-like properties, C75 treatment efficiently reduced expressions of stem-related genes ALDH1 and OCT4 in tumor spheres (Figure 7, A and B) and persistently inhibited sphere formations of such CSCs (Figure 7, C and D). C75 also delayed tumorigenicity and inhibited tumor outgrowth of CSCs in NSG mice (Figure 7, E and F). The delayed tumor growth by C75 was OPA1 dependent, as overexpression of OPA1 significantly blocked the inhibitory effect of C75 (Figure 7G). Such findings were confirmed with the genetic knockdown of FASN in tumor spheres. We found that FASN knockdown in tumor spheres inhibited their expressions of ALDH1 and OCT4 (Figure 7H), accompanied by impaired stem properties of such CSCs (Figure 7I). Finally, experiments with a second FASN siRNA and the reintroduction of FASN by overexpression confirmed the crucial role of FASN in controlling expressions of stem-related genes (Figure 7J). In contrast, inhibition of CPT1 using Etomoxir exerted no significant effect on expressions of the stem-related genes in CSCs (Figure 7K). In collective, high lipogenesis accounts for high OPA1 in CSCs, licensing mitochondrial-fusion^hi^ and stem-like properties.

### Lipogenesis^hi^ acts via transcription factor SPDEF to induce high OPA1.

**Figure 8:**
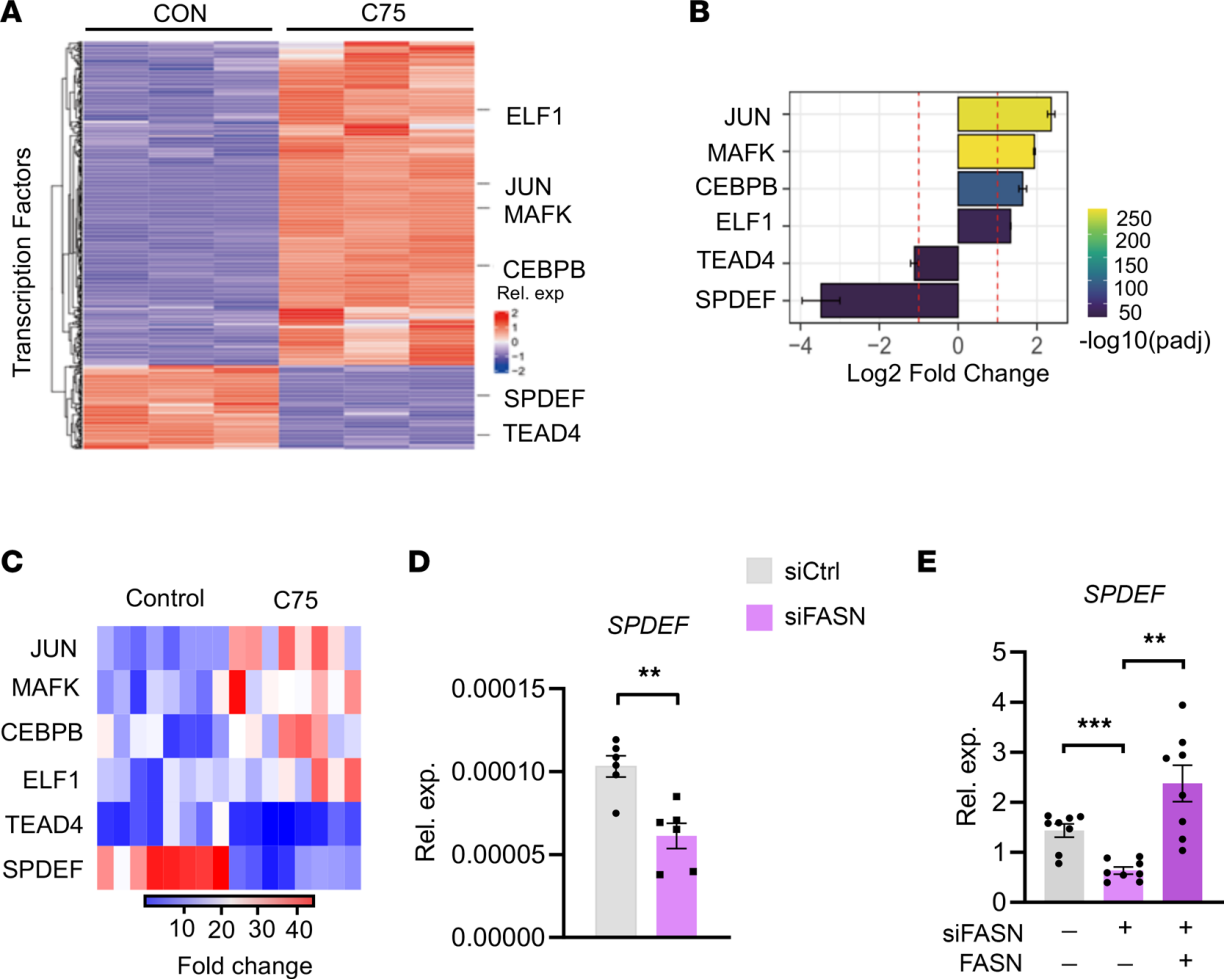
Lipogenesis promotes the expression of SPDEF for induction of high OPA1. (**A**) A549 tumor spheres with or without C75 (50 μM) treatment were analyzed for OPA1-targeting transcription factors using RNA-Seq. Data from 3 samples in each group. (**B**) Histogram showing the degree of changes in JUN, MAFK, CEBPB, ELF1, TEAD4, and SPDEF in A549 tumor spheres ± C75 (50 μM) treatment. RNA-Seq from 3 samples in each group. (**C**) A549 tumor sphere cells ± C75 (50 μM) treatment were analyzed for the indicated genes’ mRNA expressions by qPCR. Heatmap from 8 independent experiments. (**D**) A549 tumor spheres were transfected with FASN siRNA or the control and detected for mRNA expressions of SPDEF after 12 hours. Mean ± SEM from 6 independent experiments. (**E**) Experiments using a second FASN siRNA and the reintroduction of FASN confirmed the crucial role of FASN in regulating SPDEF expression in A549 tumor spheres. Mean ± SEM from 8 independent experiments. Each dot represents data from 1 experiment. **P < 0.01, ***P < 0.001 with paired *t* test (**D**) and ANOVA plus Tukey’s method (**E**).

To explore how lipogenesis^hi^ induced OPA1 expression in CSCs, tumor spheres with or without C75 treatment were subjected to RNA-Seq and transcriptomic profiling, which resulted in plenty of changes in gene expressions (Figure 8A). When cross-matched with transcription factors that can bind to OPA1 promoter in GTRD Database (41), we uncovered 6 transcription factors including JUN, MAFK, CEBPB, ELF1, TEAD4, and SPDEF (Figure 8, A and B). Of note, SPDEF, a member of the E26 transformation-specific (ETS) family, exerted the most apparent changes upon C75 treatment of tumor spheres (Figure 8B). We confirmed such RNA-Seq data using qPCR and verified that SPDEF was reduced at the most significant level in response to C75 (Figure 8C). Furthermore, genetic knockdown of FASN efficiently reduced SPDEF expression in tumor spheres (Figure 8D). Experiments using a second FASN siRNA and the reintroduction of FASN also confirmed the essential role of FASN in regulating SPDEF expressions (Figure 8E).

**Figure 9:**
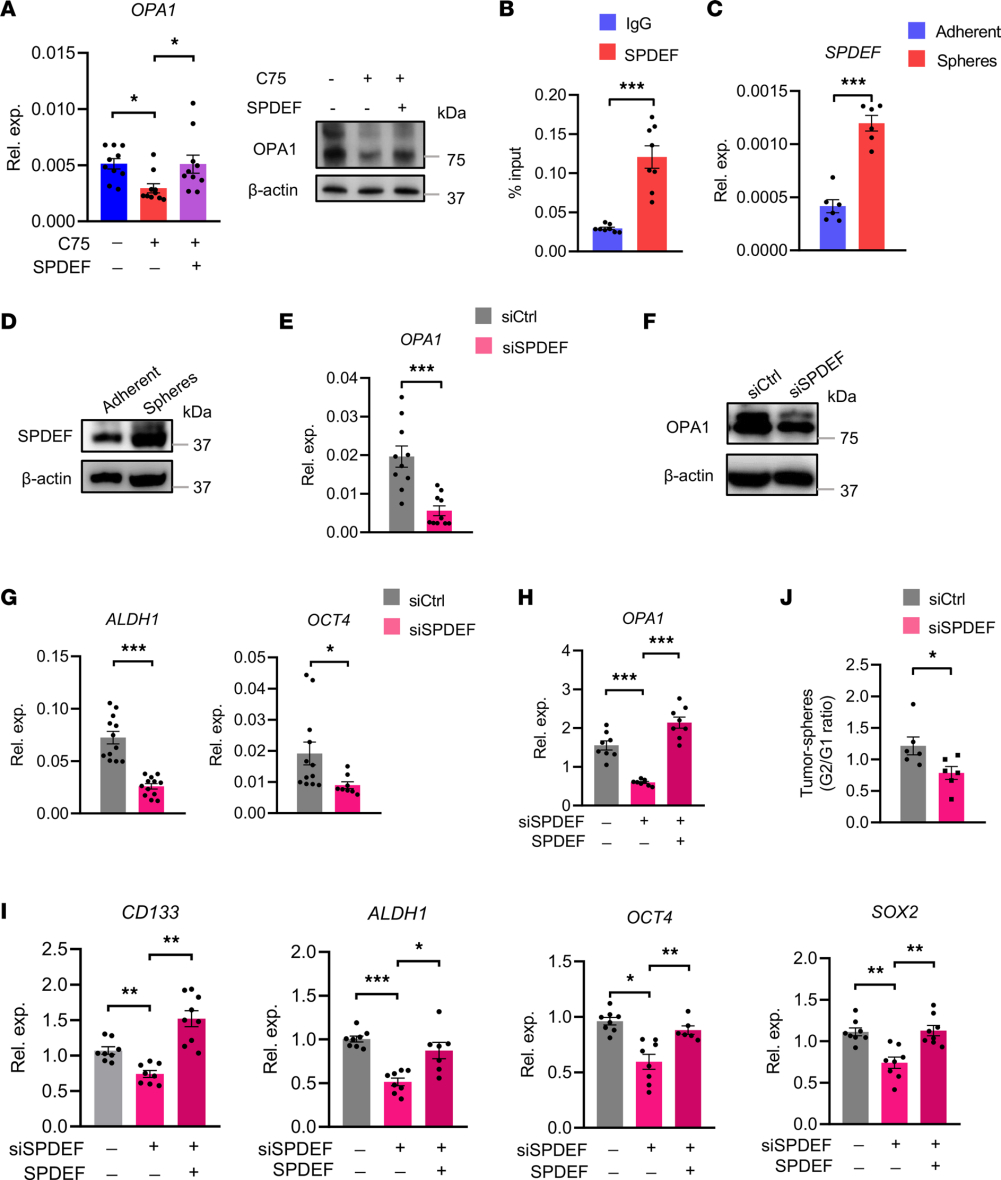
SPDEF drives the transcription of OPA1 in CSCs. (**A**) A549 tumor spheres with or without SPDEF overexpression were treated in the presence or absence of C75 (50 μM) and were monitored for OPA1 expressions. Representative and mean ± SEM from 5–10 independent experiments. (**B**) OPA1 promoter occupancy by SPDEF determined by ChIP and qPCR in A549 tumor spheres. Mean ± SEM from 8 independent experiments. (**C** and **D**) A549 tumor spheres and adherent cells were detected for expression of SPDEF by qPCR and immunoblots. Representative and mean ± SEM from 6 independent experiments. (**E** and **F**) A549 tumor spheres with or without SPDEF knockdown were detected for OPA1 expression by either qPCR or immunoblot. Representative and mean ± SEM from 10 independent experiments. (**G**) A549 tumor spheres with or without SPDEF knockdown were detected for ALDH1 and OCT4 expression by qPCR. Mean ± SEM from 12 independent experiments. (**H** and **I**) Experiments using a second SPDEF siRNA and the reintroduction of SPDEF by overexpression confirmed the crucial role of SPDEF in regulating expressions of OPA1 and stem-related genes in A549 tumor spheres. Mean ± SEM from 8–9 independent experiments. (**J**) The G1 and G2 SPDEF–depleted A549 tumor spheres were analyzed for sphere formations. Mean ± SEM from 6 independent experiments. *P < 0.05, **P < 0.01, ***P < 0.001 with paired *t* test (**B**–**G** and **J**) and ANOVA plus Tukey’s method (**A**, **H**, and **I**).

To detect whether SPDEF was involved in lipogenesis-induced OPA1 expression, tumor spheres were transfected with a SPDEF vector to achieve SPDEF overexpression (Supplemental Figure 5A), followed by C75 treatment and OPA1 analyses. We found that SPDEF overexpression could block the inhibitory effect of C75 on OPA1 expression (Figure 9A). ChIP assay demonstrated that SPDEF could bind to the OPA1 gene (Figure 9B). These data regard a responsible role of SPDEF in lipogenesis-induced OPA1 expression in CSCs.

To confirm the function of SPDEF in driving OPA1 expression, tumor spheres were manipulated for SPDEF expression and detected for OPA1 mRNA level. In line with the high OPA1 in CSCs, SPDEF was expressed at a higher level in tumor spheres (Figure 9, C and D). Genetic knockdown of SPDEF using siRNA efficiently reduced OPA1 expressions in tumor spheres (Supplemental Figure 5B and Figure 9, E and F), supporting SPDEF-instructed high OPA1 in CSCs. Furthermore, SPDEF knockdown reduced expressions of stem-related genes, including ALDH1 and OCT4 (Figure 9G). Experiments with a second SPDEF siRNA and the reintroduction of SPDEF confirmed that SPDEF was crucial for licensing expressions of OPA1 and stem-related genes in CSCs (Figure 9, H and I). Accordingly, the genetic knockdown of SPDEF persistently impaired the sphere formation ability of tumor spheres (Figure 9J). SPDEF is critically involved in lipogenesis-induced OPA1 expression, driving high OPA1 and stem-like properties of CSCs.

### CSCs derived from patients with NSCLC exert lipogenesis^hi^-induced high OPA1 signaling.

To evaluate the clinical relevance of our findings, sections of tumor tissues derived from patients with NSCLC were stained with CD133 antibody and BODIPY. The CD133-expressing cells were predominantly PanCK^+^ (Supplemental Figure 6A), with strong accumulation of lipid droplets (Supplemental Figure 6, B and C), regarding high lipogenesis in patients’ CSCs.

**Figure 10:**
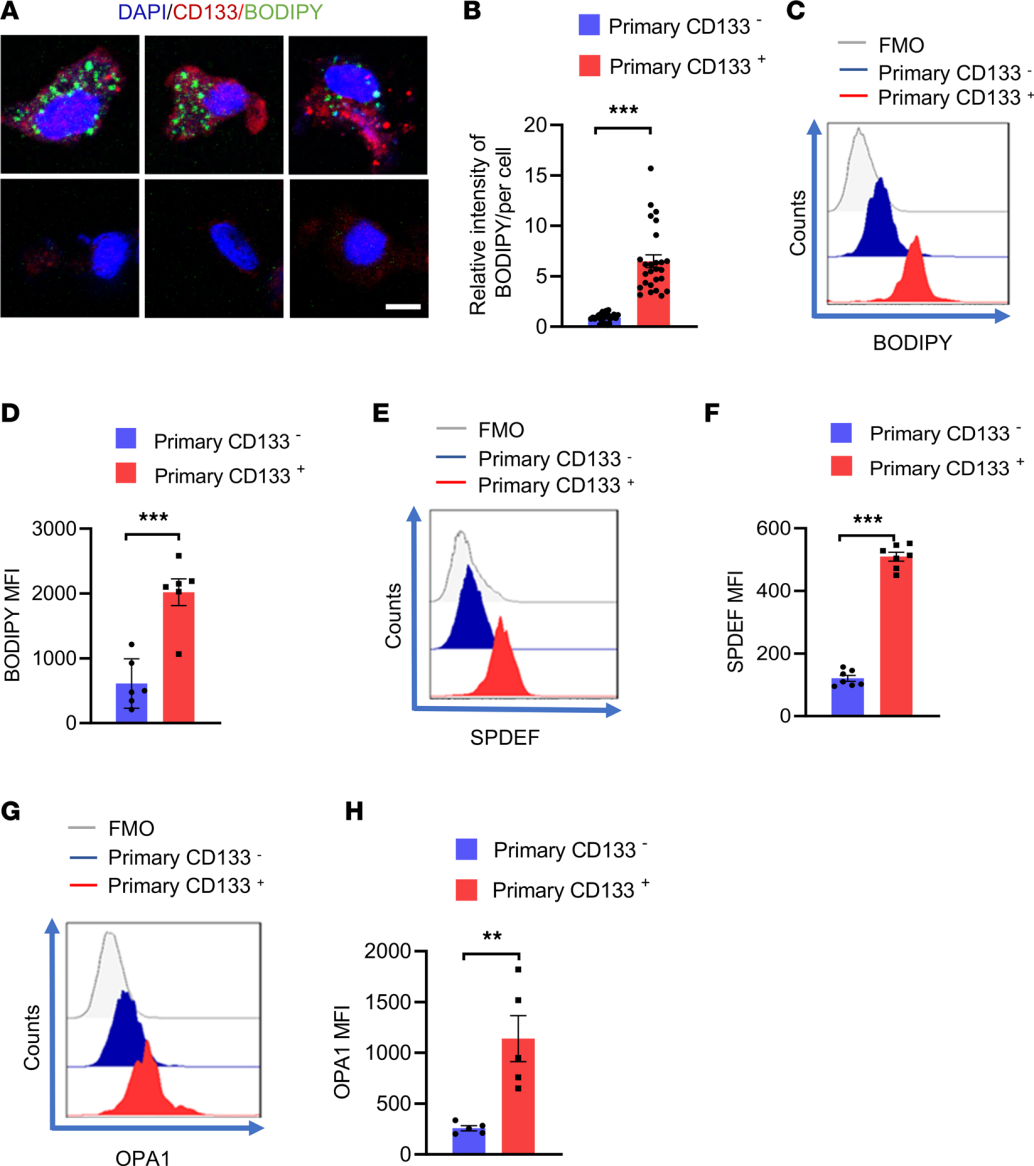
Patient-derived CSCs exert lipogenesis^hi^, SPDEF^hi^, and OPA1^hi^. (**A** and **B**) Patient-derived primary CD133^+^ and CD133^–^ tumor cells were visualized for intercellular lipid deposition with confocal microscopy. Representative and mean ± SEM from 5 independent experiments. Nuclei were stained with DAPI. Scale bar: 5 μm. (**C** and **D**) Patient-derived primary CD133^+^ and CD133^–^ tumor cells were analyzed for intracellular lipid accumulation using flow cytometry. Representative histograms and mean ± SEM from 6 independent experiments. (**E** and **F**) Patient-derived primary CD133^+^ and CD133^–^ tumor cells were analyzed for intracellular SPDEF protein levels using flow cytometry. Representative and mean ± SEM from 6 independent experiments. (**G** and **H**) Patient-derived primary CD133^+^ and CD133^–^ tumor cells were analyzed for intracellular OPA1 protein levels using flow cytometry. Shown from 5 independent experiments. Representatives from 6 independent experiments. Each dot represents data from 1 experiment. **P < 0.01, ***P < 0.001 with paired *t* test.

To overcome the limited access to patient-derived tumor cells and to directly detect the lipogenesis activity in patient-derived CSCs, organoids (PDOs) derived from patients with NSCLC were established, followed by analyses of CD133-expressing tumor cells. We performed immunostainings of PanCK and CD31, as well as H&E staining, to confirm the successful establishment of PDOs (Supplemental Figure 7). After that, CD133^+^ and CD133^–^ tumor cells from PDOs were analyzed for accumulation of lipid droplets using confocal imaging and flow cytometry. We consistently found that CD133^+^ cells exerted high lipogenesis, showing substantial accumulation of lipid droplets (Figure 10, A–D). Of note, CD133^+^ cells showed higher expressions of SPDEF and OPA1 than the CD133^–^ cells (Figure 10, E–H). These findings demonstrate high lipogenesis, high SPDEF, and high OPA1 metabolic adaptions in patients’ CSCs.

**Figure 11:**
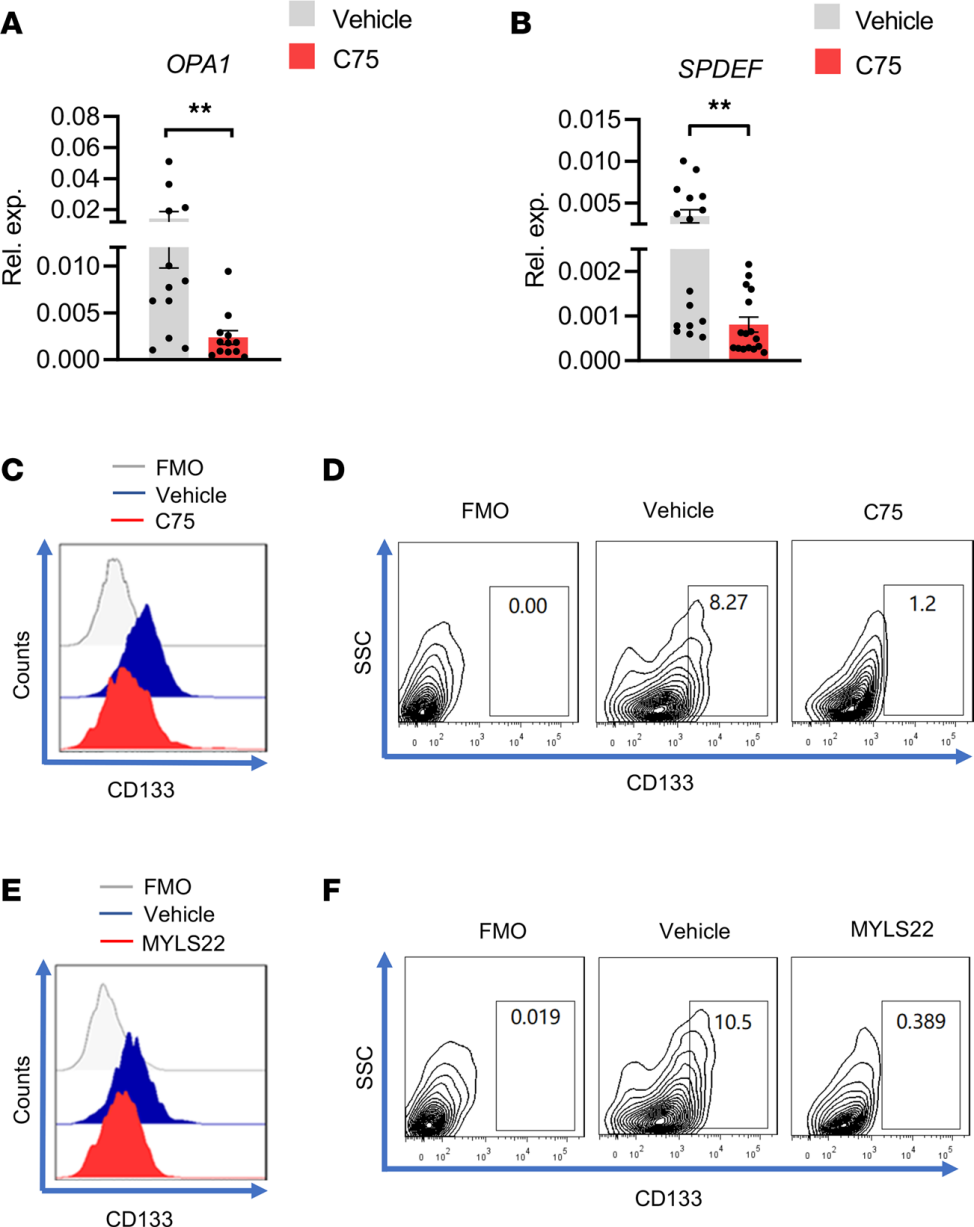
Lipogenesis-SPDEF-OPA1 signaling in patient-derived CSCs. (**A** and **B**) PDOs were treated with or without C75 (50 μM) for 12 hours and were monitored for mRNA expressions of OPA1 and SPDEF by qPCR. Mean ± SEM from 12–16 PDOs in each group. (**C** and **D**) PDOs with or without C75 (50 μM) treatment were analyzed for CD133 protein and CD133-expressing tumor cells. Representatives from 6 independent experiments. (**E** and **F**) PDOs with or without MYLS22 (40 μM) treatment were analyzed for CD133 protein and CD133-expressing tumor cells. Representatives from 6 independent experiments. **P < 0.01 with paired *t* test.

To determine whether high lipogenesis attributed to high SPDEF and high OPA1 in patient-derived CSCs, PDOs were treated with C75 and were analyzed for expressions of SPDEF plus OPA1. We found that the C75 treatment significantly reduced the expressions of SPDEF and OPA1 in PDOs (Figure 11, A and B). The C75 treatment of PDOs decreased CD133-expressing cells in PDOs (Figure 11, C and D), and treatment of PDOs with MYLS22 also minimized CD133-expressing cells (Figure 11, E and F). In essence, patient-derived CSCs exert aberrant lipogenesis, licensing high SPDEF and high OPA1 for stem maintenance.

### Targeting lipogenesis and mitochondrial fusion restricted the CSC pool and outgrowth of PDOs.

**Figure 12:**
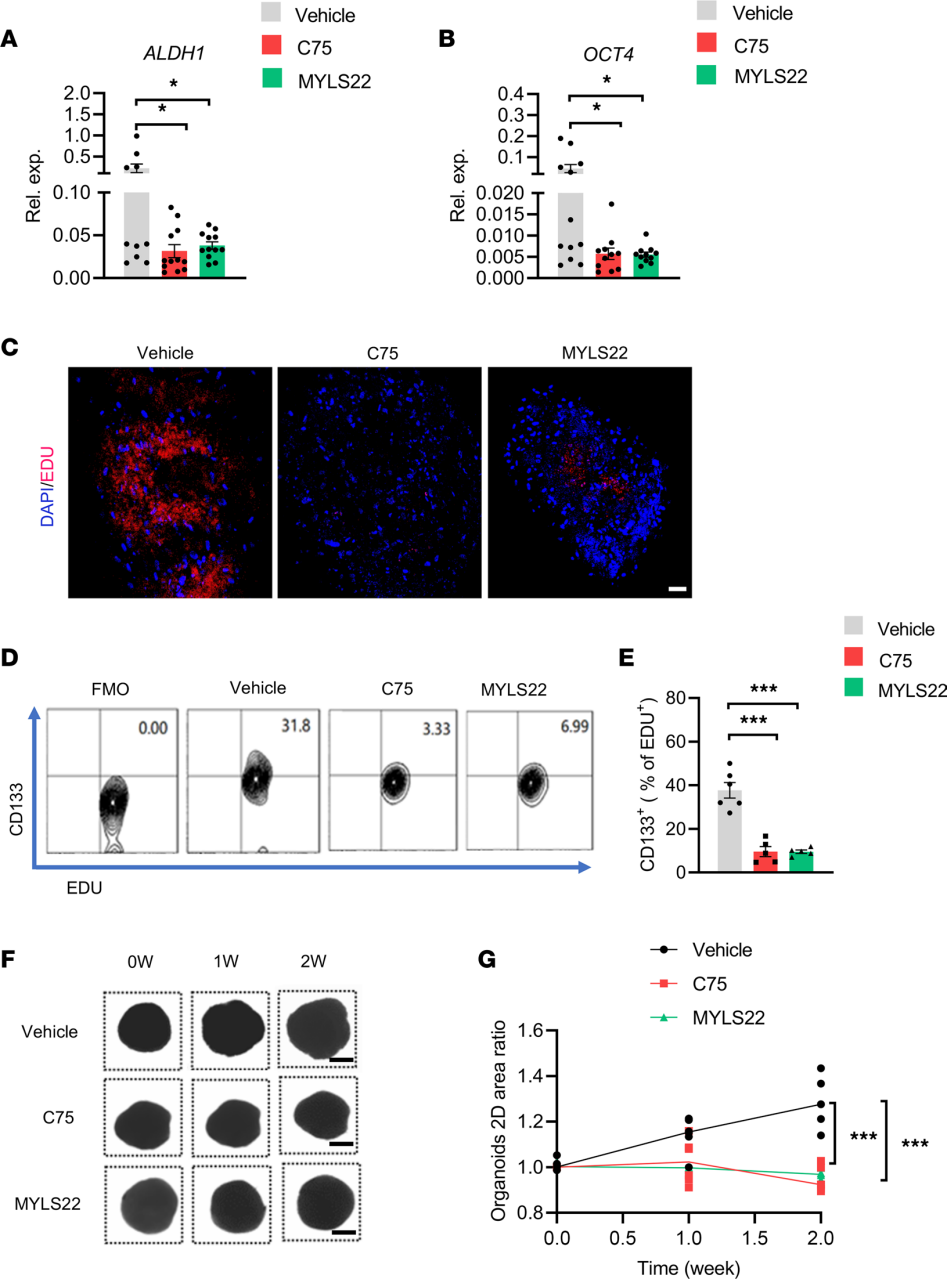
Targeting lipogenesis and mitochondrial fusion restrict CSCs in PDOs. (**A** and **B**) PDOs were treated with or without C75 (50 μM) and MYLS22 (40 μM) for 7 days and tested for mRNA expression of CSC-related genes ALDH1 plus OCT4. Mean ± SEM from 12 PDOs in each group. (**C**) PDOs were treated with or without C75 (50 μM) and MYLS22 (40 μM) for 7 days and monitored for EdU^+^ proliferating tumor cells. Nuclei were stained with DAPI. A representative from 6 PDOs in each group. Scale bar: 20 μm. (**D** and **E**) PDOs were treated with or without C75 (50 μM) and MYLS22 (40 μM) for 7 days and monitored for CD133^+^ cells within EdU^+^ proliferating cells. Representative and mean ± SEM from 6 independent experiments. (**F** and **G**) PDOs were treated with C75 (50 μM) and MYLS22 (40 μM) for the indicated time and analyzed for organoid growth by measuring 2D areas. Shown are sample bright-field images of individual PDOs. Scale bars: 500 mm. Data from 5 PDOs in each group. *P < 0.05, ***P < 0.001 with ANOVA and Tukey’s method.

PDOs have shown a spectrum of responses to conventional and investigational drugs, and these responses essentially mimic the reaction of those patients to the same agents (42, 43). To evaluate the translational application of lipogenesis and mitochondrial fusion–based therapeutics in NSCLC treatment, NSCLC PDOs were treated with either lipogenesis inhibitor C75 and mitochondrial fusion inhibitor MYLS22. Both C75 and MYLS22 could minimize the expression of stem-related genes ALDH1 and OCT4 of PDOs (Figure 12, A and B). Furthermore, C75 and MYLS22 efficiently reduced the proliferating EdU^+^ cells in PDOs (Figure 12C). Notably, such treatments with C75 and MYLS22 inhibited the expansion of patient-derived CSCs, as evidenced by decreased frequency of CD133^+^ cells within proliferating EdU^+^ ones (Figure 12, D and E). Finally, C75 and MYLS22 efficiently impaired the outgrowth of NSCLC PDOs (Figure 12, F and G). These findings assign lipogenesis and mitochondrial fusion promising therapeutic targets for NSCLC management.

## Discussion

As a major obstacle in cancer management, CSCs are phenotypically, functionally, and metabolically distinguishable from non-CSC tumor cells (14). Here, we identify high OPA1 as a gatekeeper for high mitochondrial fusion activity in CSCs of human lung cancer. The aberrant lipogenesis in NSCLC CSCs instructs OPA1 transcriptome through transcription factor SPDEF. Accordingly, blocking lipogenesis and mitochondrial fusion activity can inhibit CSC expansion and outgrowth of PDOs, providing mechanism-based therapeutic strategies for NSCLC treatment.

Mitochondria are dynamic organelles with a variety of morphologies controlled by processes of fusion and fission, and they have emerged as being crucial to the maintained function of such organelles and vital for tissue homeostasis (44, 45). The dynamic properties of mitochondria are critical for their optimal function in energy generation, as optimal mitochondrial oxidative phosphorylation relies on mitochondrial fusion for high-quality mitochondria and mitochondrial fission to eliminate damaged mitochondria (46). The critical components of the machinery mediating mitochondrial fusion and fission belong to the dynamin family of GTPases that utilize GTP hydrolysis to drive mechanical work on biological membranes (27). While mitochondrial fusion requires 3 large GTP-hydrolyzing enzymes, including MFN-1/2 and OPA1, DRP1 plays a central role in mitochondrial fission (47). Mitochondrial fission begins with an interaction of mitochondria with the endoplasmic reticulum (48). Subsequently, DRP1 translocates from cytosolic onto the mitochondria, assembles into spiral structures, and constricts mitochondrial tubules for fission (49). Accumulating studies have reported that mitochondria are highly fragmented in cancer cells, exerting lower expression of MFN2 and higher expression of DRP1 (25). Given the similarities between stem cells and cancer cells in terms of replication, they have similar metabolic features, primarily relying on aerobic glycolysis to generate ATP (50). Thus, mitochondrial fission is believed to be crucial for the development and function of stem cells (30, 51). Indeed, brain tumor CSCs exert fragmented mitochondria compared with non-CSCs, activating phosphorylation of DRP1 in CSCs and inhibitory phosphorylation of DRP1 in non-CSCs (52). Targeting BRD4 by genetic knockdown or chemical inhibitors blocked mitochondrial fission and consequently caused CSC exhaustion and loss of tumorigenic capability of prostate CSCs (53). However, CSCs are metabolically reprogrammed and distinguishable from non-CSC tumor cells, primarily relying on mitochondrial oxidative phosphorylation for energy generation (54–57). While NSCLC CSCs maintain a high quality of mitochondria by removing dysfunctional mitochondria through mitophagy (26), here we uncovered a high mitochondrial fusion activity in NSCLC CSCs relative to non-CSCs and pinpointed high OPA1 as the central mediator for stem properties. As mitochondrial fusion often correlates with high mitochondrial oxidative phosphorylation (58), our findings might at least partly explain how CSCs rely on mitochondria for stemness. Essentially, NSCLC CSCs rely on increased mitophagy to remove the damaged mitochondria and robust mitochondrial fusion of the remaining mitochondria to optimize the mitochondrial metabolism for maintaining stem-like properties. Similarly, *Drosophila* brain tumors also contain a rapidly dividing stem cell population that relies on oxidative phosphorylation and mitochondrial fusion for tumorigenesis (35). Increased mitochondrial fission by knockdown of MFN1 promotes the survival of hepatocellular carcinoma cells both in vitro and in vivo, mainly by facilitating autophagy and inhibiting mitochondria-dependent apoptosis (59).

SPDEF is localized to chromosome 6p21.31, encodes a 335 aa protein, preferentially binds the target gene through the high-affinity sequence of GGAT, and is frequently reported to play a suppressive function in tumor progression and metastasis (60). In studies of mouse and human colorectal cancer, SPDEF induces a quiescent state of cancer cells by disrupting the binding of β-catenin to TCF1 and TCF3 and by regulating genes that control the cell cycle (61). SPDEF also functions as a tumor suppressor in prostate cancer by inhibiting mRNA and protein levels of Foxm1, a transcription factor critical for tumor cell proliferation (62). However, SPDEF plays a diverse role in the expression levels, clinicopathologic importance, biological function, and prognostic evaluation in breast cancer, which mainly depends on different subtyping of breast cancer (63); these processes distinct expressions and functions of SPDEF in different types of tumor cells. Here we reveal SPDEF as crucial for stem-like properties of human NSCLC CSCs. In NSCLC tumor spheres, SPDEF binds to the OPA1 gene, promoting OPA1 expression, mitochondrial fusion activity, and stem-like properties. Similarly, SPDEF-mediated microRNA-448 activation is also involved in the oncogenicity and self-renewal of hepatocellular carcinoma stem cells (64). However, the precise functions of SPDEF in different cancers and tumor cell subsets still deserve successive studies.

Lipids are essential components of cell and organelle membranes, and fatty acids are well acknowledged to be required for CSC maintenance (65–67). Specifically, alterations in lipid metabolism satisfy the energy demands and biomass production of CSCs and contribute to the activation of several crucial oncogenic signaling pathways, including Wnt and Hippo/YAP signaling (67–69). In the current study, we identify lipogenesis^hi^ as an initiator for SPDEF^hi^, high OPA1, and mitochondrial-fusion^hi^ in NSCLC CSCs, providing a mechanism underlying the crucial involvement of lipid metabolism in CSC biology. In support, FASN, a rate-limiting enzyme for de novo lipogenesis, is consistently found to facilitate in CSCs (70). A comparison of lipidomic profiles between CSCs and non-CSCs also suggests that monounsaturated fatty acids affect the formation and stemness of CSCs (70, 71). Therefore, ovarian CSCs require more monounsaturated fatty acids than their nonstem counterparts (72). Mechanistically, cell membrane fluidity highly depends on the degree of lipid unsaturation, and low membrane fluidity inhibits metastasis and stemness in cancers (70, 73). Furthermore, lipid droplets, which are cytoplasmic organelles for fatty acids storage, are reported to be closely associated with tumor aggression and a distinctive marker for CSCs (74–76). Although molecular mechanisms underpinning lipogenesis^hi^ in CSCs remain unclear, one possibility might be the Notch1^hi^ feature of CSCs, which could drive mTORC1 activity and subsequent lipogenesis (77–79). However, which kind of lipids was responsible and how such lipid acids instruct SPDEF^hi^ in CSCs still remain to be elucidated.

In this study, human NSCLC cell line A549 was dominantly used to identify how lipogenesis drives mitochondrial fusion for stem maintenance of CSCs. We have also extended the main findings to another NSCLC cell line, Calu-1, which fundamentally reproduced the phenomenon of lipogenesis-induced SPDEF-OPA1 signaling in stem-like cells (Supplemental Figure 8). Of note, we confirmed the crucial function of the lipogenesis-SPDEF-OPA1 pathway in licensing CSC stemness using primary cells from PDOs. However, we would like to acknowledge some limitations. One is that the current patient cohort for PDO data is relatively small, and a large number of the patient cohort would substantiate whether the PDO data firmly validate the overall concept. Another is that CSCs have been shown to transition between different states, and there are likely multiple CSCs in different NSCLC types (14, 80). Our findings might focus on the metabolically highly active and proliferative cells rather than quiescent CSCs. Successive research exploring mechanisms underpinning the CSC transitions is required to pave the way for translational studies.

In summary, NSCLC CSCs exert high OPA1, promoting mitochondrial fusion activity for CSC maintenance. Mechanistically, CSCs have robust lipogenesis, resulting in OPA1 overexpression via transcription factor SPDEF. Such a lipogenesis-triggered SPDEF-OPA1 axis holds well in CSCs derived from patients with NSCLC. Accordingly, targeting lipogenesis and mitochondrial fusion effectively controls the CSC pool in NSCLC PDOs. Thus, OPA1 is a gatekeeper that connects lipid metabolism and mitochondrial dynamics for CSC maintenance in human lung cancer.

## Methods

### Patients.

Patients with a diagnosis of NSCLC were recruited, while those with other uncontrolled medical or inflammatory diseases were excluded. Patient characteristics, including sex, age, TNM stages, and histological types, were summarized in Supplemental Table 2.

### PDO.

PDOs were established and cultured as previously described (81). Briefly, lung cancer tissues were minced into approximately 0.5–1 mm diameter pieces using fine dissection scissors (Fine Science Tools). Tumor pieces were incubated with RBC lysis buffer (Solarbio, R1010) under gentle rotation for 5 minutes at room temperature to lyse contaminating RBCs. Then, tumor pieces were distributed in ultra-low attachment 6-well culture plates (Corning) with 4 mL per well of PDO medium containing 50% DMEM/F12 (Corning, 10-092-cv), 50% Neurobasal (Thermo Fisher Scientific, 21103049), 1***×*** GlutaMax (Thermo Fisher Scientific, 35050061), 1***×*** NEAAs (Thermo Fisher Scientific, 11140050), 1***×*** PenStrep (Beyotime, C0222), 1***×*** N2 supplement (Thermo Fisher Scientific, 17502048), 1***×*** B27 w/o vitamin A supplement (Thermo Fisher Scientific, 12587010), 1Cat. 2-mercaptoethanol (Thermo Fisher Scientific, 21985023), and 2.5 mg/mL human insulin (Beyotime, P3376-100IU) and placed on an orbital shaker rotating at 120 rpm within a 37°C, 5% CO_2_, 90% humidity sterile incubator. PDOs were accessed by immunostainings of PanCK and CD31, together with H&E staining.

### Tumor spheres and adherent cells.

Human NSCLC cell line A549 from ATCC was maintained in DMEM (Corning) supplemented with 10% FBS (MilliporeSigma) and 50 units/mL penicillin/streptomycin (Beyotime). Tumor spheres were enriched using DMEM/F12 (Corning, 10-092-cv) supplemented with 1***×*** B-27 (Thermo Fisher Scientific), EGF (20 ng/mL; Novoprotein, C029), bFGF (20 ng/mL; Novoprotein, C046), insulin (Beyotime, P3376-100IU), and penicillin/streptomycin (50 units/mL, Beyotime, C0222) (37). For serial passaging, spheres were harvested after 6 days using a 40 mm cell strainer, dissociated into single cells with continuous pipetting, and then regrown in the same conditions. For the sphere formation assay, the number of spheres with > 40 mm diameters was determined using a CASY Cell Counter (BioCentury).

### Transfection and RNA interference.

For siRNA transfections, cells (2 ***×*** 10^5^ cells per well) were placed in a 24-well plate (Corning) and transfected with specific siRNAs (100 nM; Santa Cruz Biotechnology Inc.) using lipofectamine stem (Thermo Fisher Scientific, STEM00001). All reagents were used according to the manufacturer’s instructions.

### Real-time PCR.

Total mRNA was extracted from cells by phenol/chloroform extraction. Total RNAs were converted to cDNA using All-in-One 1st Strand cDNA Synthesis SuperMix (Novoprotein). qPCR was carried out using SYBR Super mix (Novoprotein). Cycling conditions were as follows: 95°C for 2 minutes, 95°C for 5 seconds, and 60°C for 34 seconds, cycled 40 times. The relative quantity of mRNA was expressed as 2^–ΔCt^. Sequences of the primers were listed in Supplemental Table 3.

### RNA-Seq and metabolomic analyses.

RNA-Seq was performed by the GIGA Genomics Facility (BioNovoGene). Tumor spheres and adherent cells, as well as tumor spheres ± C75 treatment, were subjected to RNA-Seq. Metabolomic analyses of lipids in tumor spheres and adherent cells were performed by Metware Biotechnology. Those data were deposited in the National Genomics Data Center (accession no. PRJCA006881). For the RNA-Seq, 1–2 μg total RNA of each sample was taken for RNA-Seq library preparation using KAPA Stranded RNA-Seq Library Prep Kit (Illumina). Adapter sequences were trimmed from raw sequencing reads, and sequencing reads with an average quality score lower than Q20 were filtered to produce clean data. HISAT2 with default parameters was used to align clean sequencing reads to the human reference genome (82). HTSeq was used to calculate the read count expression value (83). Differential expression analysis was performed using DESeq (84). Genes with absolute fold change over 2 and adjusted *P* value lower than 0.05 were considered significant.

### Flow cytometry.

Cells from PDOs were prepared using 300 units/mL collagenase IV (Worthington, LS004210) at 37°C for 30 minutes, followed by three 5-minute washes with RPMI-1640 (Corning) supplemented with 10% FBS (MilliporeSigma) and 50 units/mL penicillin/streptomycin (Beyotime). Cells were treated with BD Fix Buffer I and Perm Buffer III and were stained for 45 minutes at room temperature with the following antibodies: PE-conjugated anti-CD133 antibody (eBiosciences, 12-1338-42), anti–human OPA1 antibody (Cell Signaling Technology, 67589), and anti–human SPDEF antibody (Santa Cruz Biotechnology Inc., sc-166846). BODIPY 493/503 (Thermo Fisher Scientific, D3922) was used to quantify the intracellular accumulation of lipid droplets. Cells were analyzed using BD Canto II FACS analysis, and for some experiments, live cells were sorted with the BD FACS Influx instrument. Fluorescence minus one (FMO) was used for the gating strategy. Data were analyzed with FlowJo software.

### TEM and mitochondrial areas.

TEM for mitochondrial fusion activity was performed as previously described (85). Cells were fixed with 2.5% glutaraldehyde buffered in 0.1M sodium cacodylate (pH 7.4). Following TEM — including postfixation with osmium tetroxide, dehydration with gradient ethanol, embedding and polymerization with epoxy resin, and ultrathin sectioning — imaging (under an FEI Tecnai12 transmission electron microscope) was applied in National Institute for Viral Disease Control and Prevention, China CDC. Tubulated and fragmented mitochondrion were defined as described previously (86). Mitochondrial areas were quantified using ImageJ (NIH) as described previously (85).

### Immunofluorescence.

Cell staining with MitoTracker Red (Thermo Fisher Scientific, M22425), CellLight Plasma Membrane-RFP (Thermo Fisher Scientific, C10505), and BODIPY 493/503 (Thermo Fisher Scientific, D3922) was performed according to manufacturer’s instructions. For immunofluorescence, fixed and permeabilized cells were incubated with primary antibodies: anti-PanCK (1:200 dilution, Abcam, ab7753) plus anti-CD133 (1:200 dilution; MilliporeSigma, ZRB1013). Secondary antibodies (Abcam; ab150113 [goat anti-mouse IgG H&L, Alexa Fluor 488], ab150077 [goat anti-rabbit IgG H&L, Alexa Fluor 488]) were used at 1:1,000 dilution. Cell nuclei were counterstained with Hoechst 33342 for 15 minutes at room temperature. For PDO staining, organoids were embedded with OTC and were sectioned (5 mm), deparaffinized, and stained for histological analysis or immunofluorescence. Images were visualized with confocal microscopy (Nikon) and deconvolved with ImageJ software (NIH).

### Immunoblot.

Immunoblot was performed using standard methods (87). The blots were analyzed using primary antibodies: anti-OPA1 (Cell Signaling Technology, 67589), and anti-SPDEF (Santa Cruz Biotechnology, sc-166846). β-Actin detected using anti–β-actin (Santa Cruz Biotechnology, sc-47778) was used as an internal control.

### ChIP.

The ChIP-PCR assay for SPDEF in tumor spheres was performed with a standard protocol (53). Specific DNA fragments were obtained, purified, and subjected to PCR analysis using a NovoNGS CUT&Tag High-Sensitivity Kit (Novoprotein). Primers used for ChIP-PCR are listed in Supplemental Table 4.

### Seahorse assay.

For the Seahorse assay, cells were seeded into a 96-well plate at 10 thousand cells per well and detected with the Seahorse XF Cell Mito Stress Test kit (Agilent) according to the instructions. The analyses were performed using the XF96 extracellular analyzer (Seahorse Bioscience) (26).

### Tumor xenografts.

Immune-deficient male and female B-NSG mice (Biocytogen) at 6 weeks of age were housed under specific pathogen–free conditions. Tumor spheres (1 ***×*** 10^5^ cells) with or without treatments were injected s.c. into the flank of NSG mice. Such NSG chimeras were randomly assigned into treatment and control groups. Tumor initiation and size were monitored, and tumor volumes were calculated using the following formula: (width × length^2^)/2.

### Data availability.

All data supporting the findings were included in the current study.

### Statistics.

Data are presented as mean ± SEM. Two-tailed Student’s *t* test was used for 2-group comparisons with the Bonferroni method to adjust multiple comparisons. One-way ANOVA with Tukey’s method was used for the comparison of more than 2 groups. All statistical analyses were conducted using PRISM 9.0 (GraphPad Software Inc.). *P* < 0.05 was considered significant.

### Study approval.

Appropriate informed consent was obtained from every human individual, and experiments involving human samples were conducted in compliance with Declaration of Helsinki. Experiments with NSG mice were performed following the ARRIVE guidelines. All the studies were approved by the Ethics Committee of Soochow University.

## Author contributions

ZW designed the study. ZL, JL, TW, WH, MZ, YW, JS, and HR conducted experiments and analyzed data. TR and LX participated in patient recruitment and data analyses. WX and TR were involved in the study design and data analyses. ZW and ZL wrote the manuscript with input from all authors.

## Supplementary Material

Supplemental data

Supplemental tables 1-4

## Version 1 02/21/2023

In-Press Preview

## Version 2 03/22/2023

Electronic publication
