# Supplementary material for: Lipogenesis promotes mitochondrial fusion and maintains cancer stemness in human NSCLC
Source: JCI Insight. 2023 Mar 22;8(6):e158429. doi: 10.1172/jci.insight.158429 (PMC10070109; doi:10.1172/jci.insight.158429)
Supplement: Supplemental data [file jciinsight-8-158429-s143.pdf]

Supplementary Figure 1

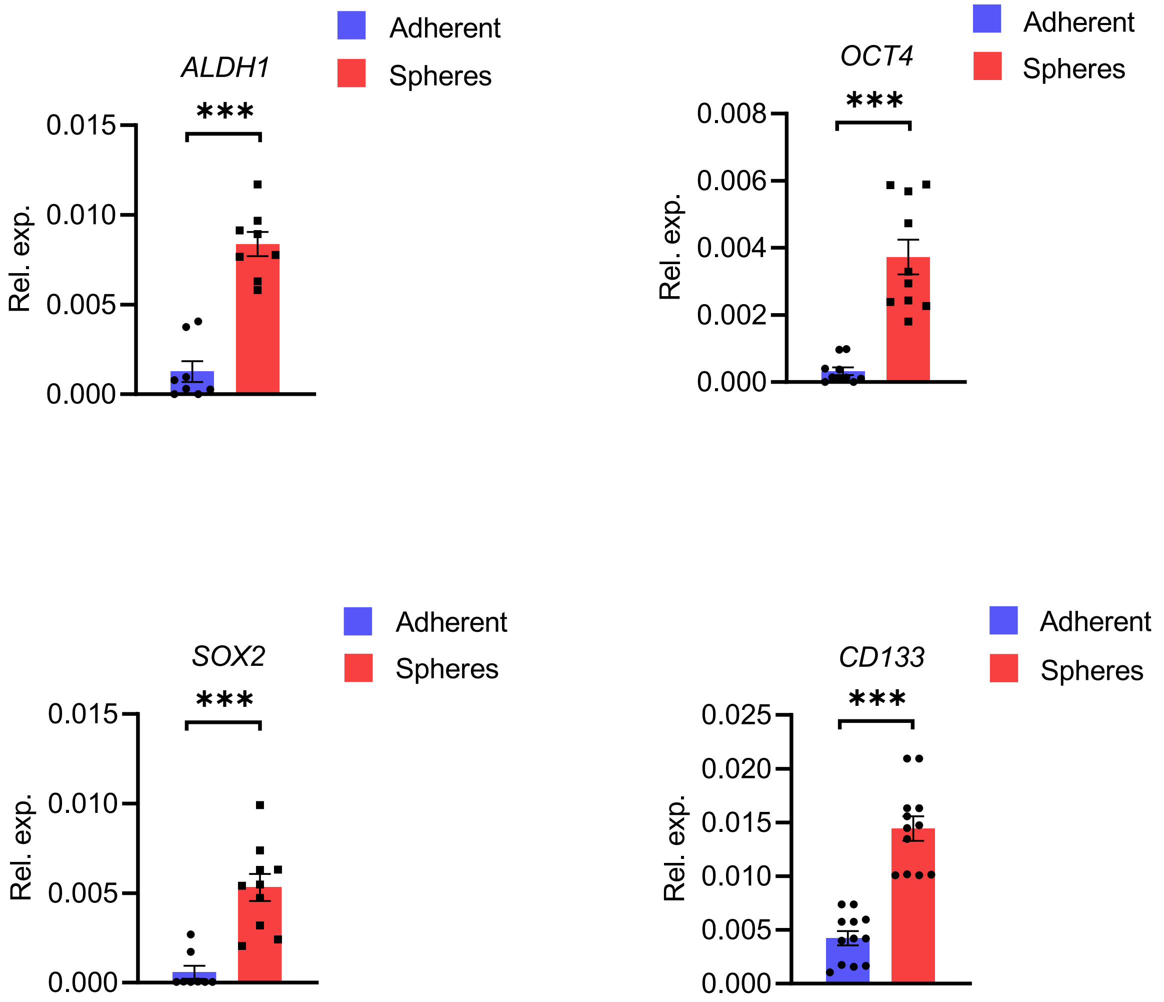

Fig. S1 Tumor-spheres are enriched with stem-like cells

Tumor-spheres and adherent cells were detected for expressions of ALDH1, OCT4, SOX2, and CD133 by qPCR. Mean±SEM from 8-12 independent experiments. ****p* < 0.001 with paired t-test.

Supplementary Figure 2

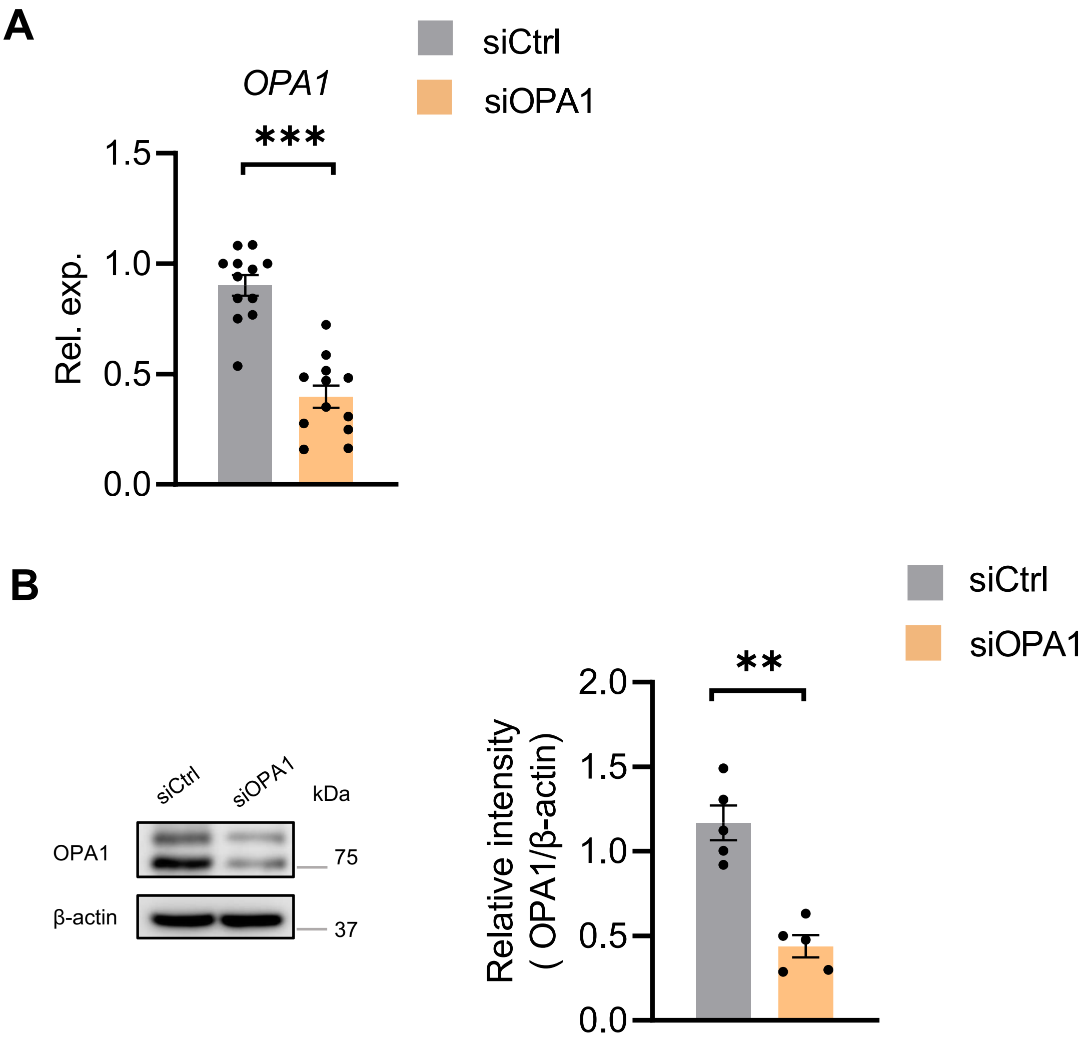

Fig. S2 Genetic knockdown of OPA1 in tumor-spheres

Tumor-spheres were transfected with OPA1 siRNA or the control siRNA. (A) mRNA expressions of OPA1 were determined using qPCR after 12 hrs. Mean±SEM from 8-12 independent experiments. (B) Protein expressions of OPA1 were detected with immunoblots after 24 hrs. Representative and mean±SEM from 5 independent experiments. ***p* < 0.01, ****p* < 0.001 with paired t-test.

Supplementary Figure 3

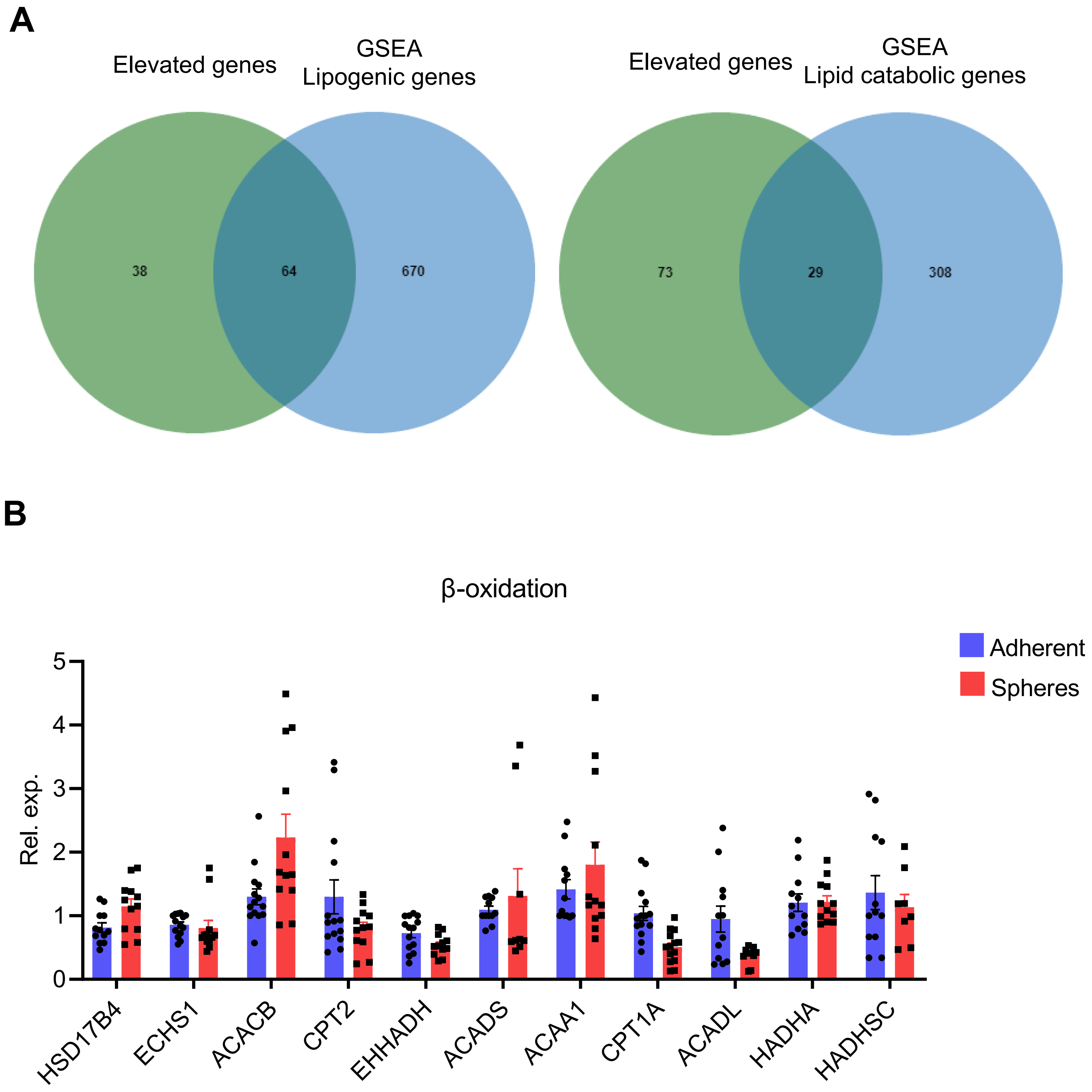

Fig. S3 Lipid β-oxidations were similar in tumor-spheres and adherent cells

(A) Lipid metabolic genes with elevated expressions in tumor spheres were dominantly involved in lipogenesis. RNA-seq from 3 samples in each group. (B) Tumor-spheres and adherent cells were detected for mRNA expressions of key enzymes involved in lipid β-oxidation by qPCR. Mean±SEM from 8-10 independent experiments. Paired t-test with Bonferroni method.

Supplementary Figure 4

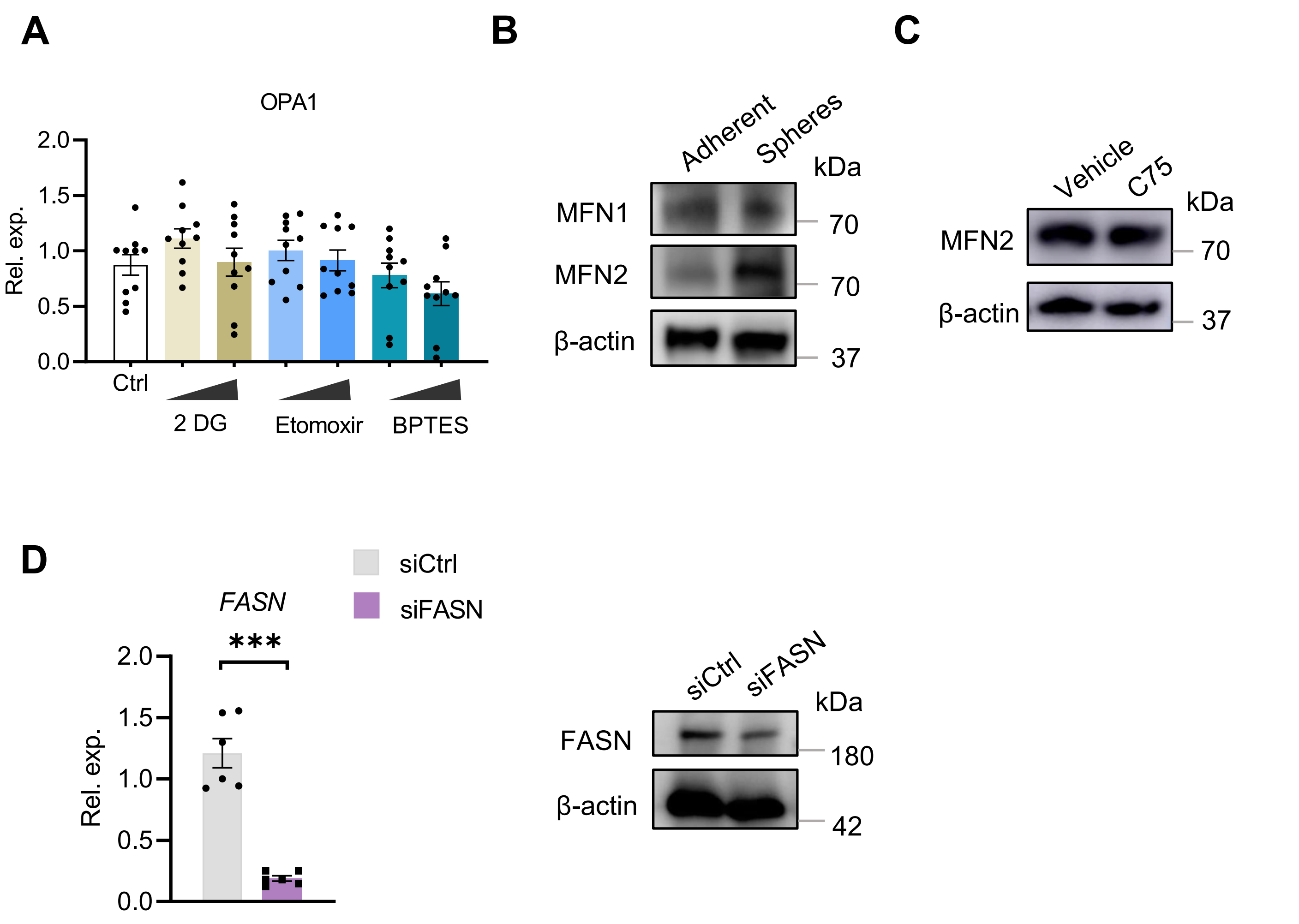

Fig. S4 Lipogenesis induces OPA1 expression of tumor-spheres

(A) Tumor-spheres were treated with an increasing dose of 2-DG (2 mM, 4mM), Etomoxir (50μM, 100 μM), and BETES (5 μM, 10 μM) for 12hrs, and analyzed for OPA1 expression with qPCR. Mean±SEM from 10 independent experiments. ANOVA with Tukey's method. (B) Protein expressions of MFN1 (ABclonal, A9880) and MFN2 (CST, #9482) in tumor spheres and adherent cells. Representatives from 3 independent experiments. (C) C75 treatment exerted no significant effect on MFN2 expression in tumor spheres. Representatives from 3 independent experiments. (D) Tumor-spheres were transected with FASN siRNA and detected for FASN expressions by qPCR and immunoblot respectively. Representative and mean±SEM from 6 independent experiments. ***p < 0.001 with paired t-test.

Supplementary Figure 5

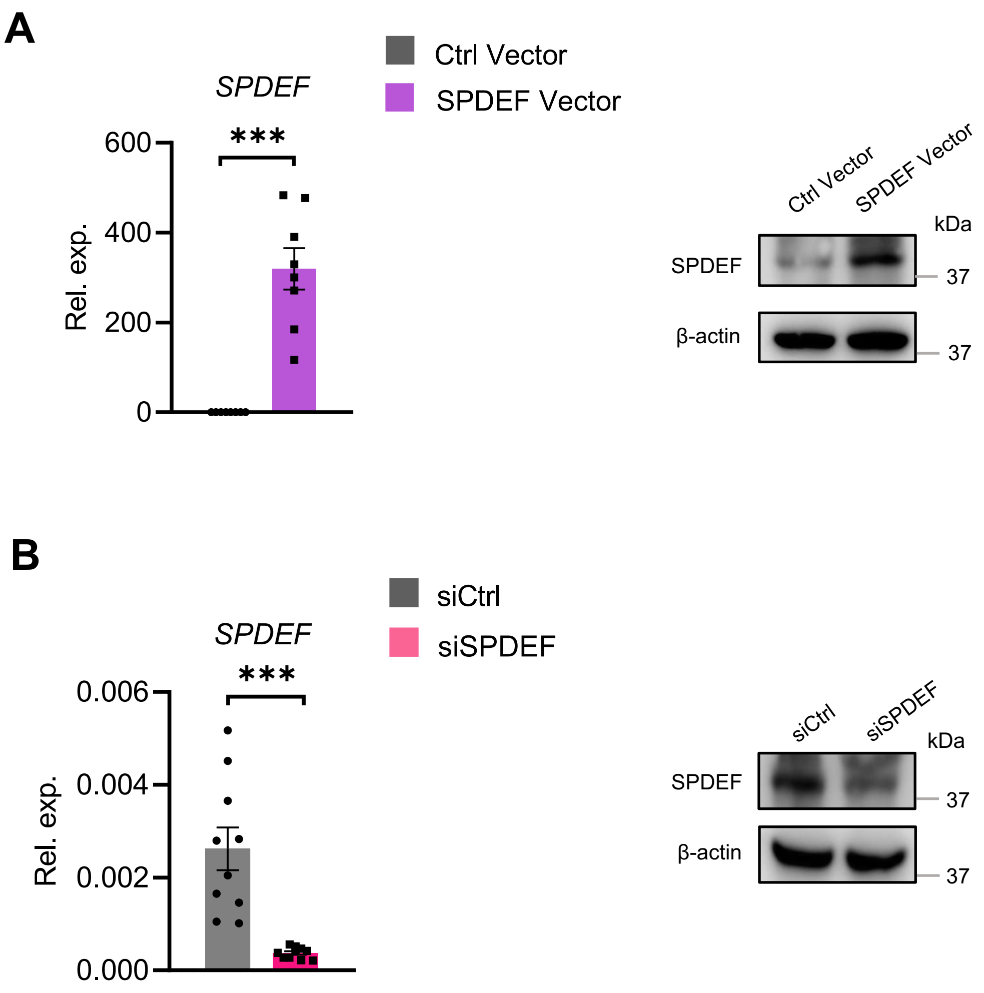

Fig. S5 Manipulation of SPDEF expression in tumor-spheres

(A) Tumor-spheres were transfected with SPDEF expression vector or control vector, and detected for SPDEF expressions by either qPCR after 12 hrs or immunoblot after 24 hrs. Representative and mean±SEM from 8 independent experiments. (B) Tumor-spheres were transfected with SPDEF siRNA or the control, and detected for SPDEF expressions by either qPCR after 12 hrs or immunoblot after 24 hrs. Representative and mean±SEM from 10 independent experiments. ***p < 0.001 with paired t-test.

Supplementary Figure 6

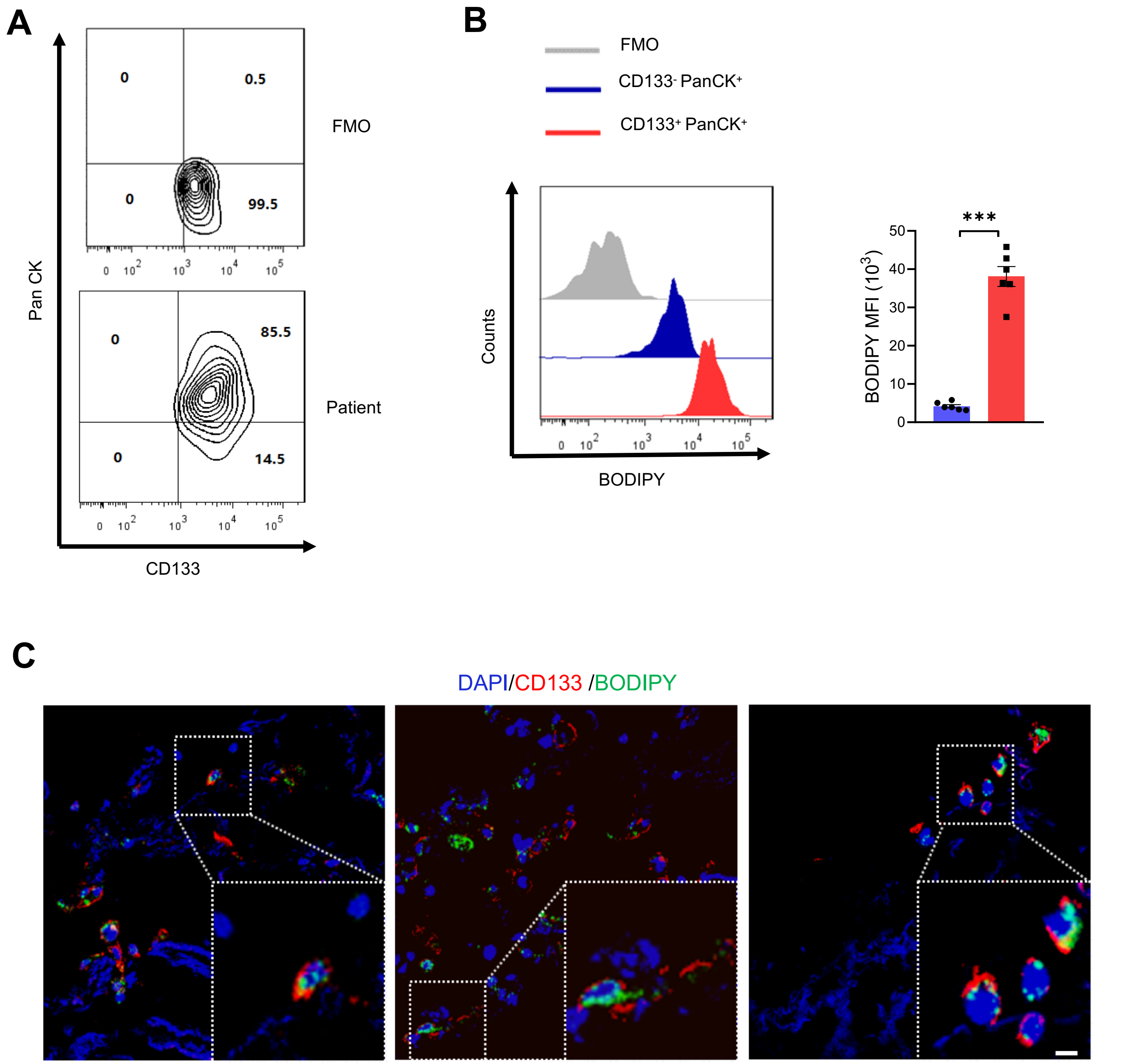

Fig. S6 Aberrant lipid deposition of CSCs in patient-derived tumor tissues

(A-B) CD133-expressing cells from tumor tissues were predominantly PanCK-positive, exerting robust accumulation of lipid droplets. Representative and mean±SEM from 6 patients. ****p* < 0.001 with paired t-test. (C)Tumor sections of NSCLC patient-derived tissues were stained with anti-human CD133 and BODIPY. Nuclei were stained with DAPI. Lipid deposition of $CD133^{+}$ CSCs was visualized with confocal microscope. Three representative images from 5 patients. Scale bar, 5 μm.

Supplementary Figure 7

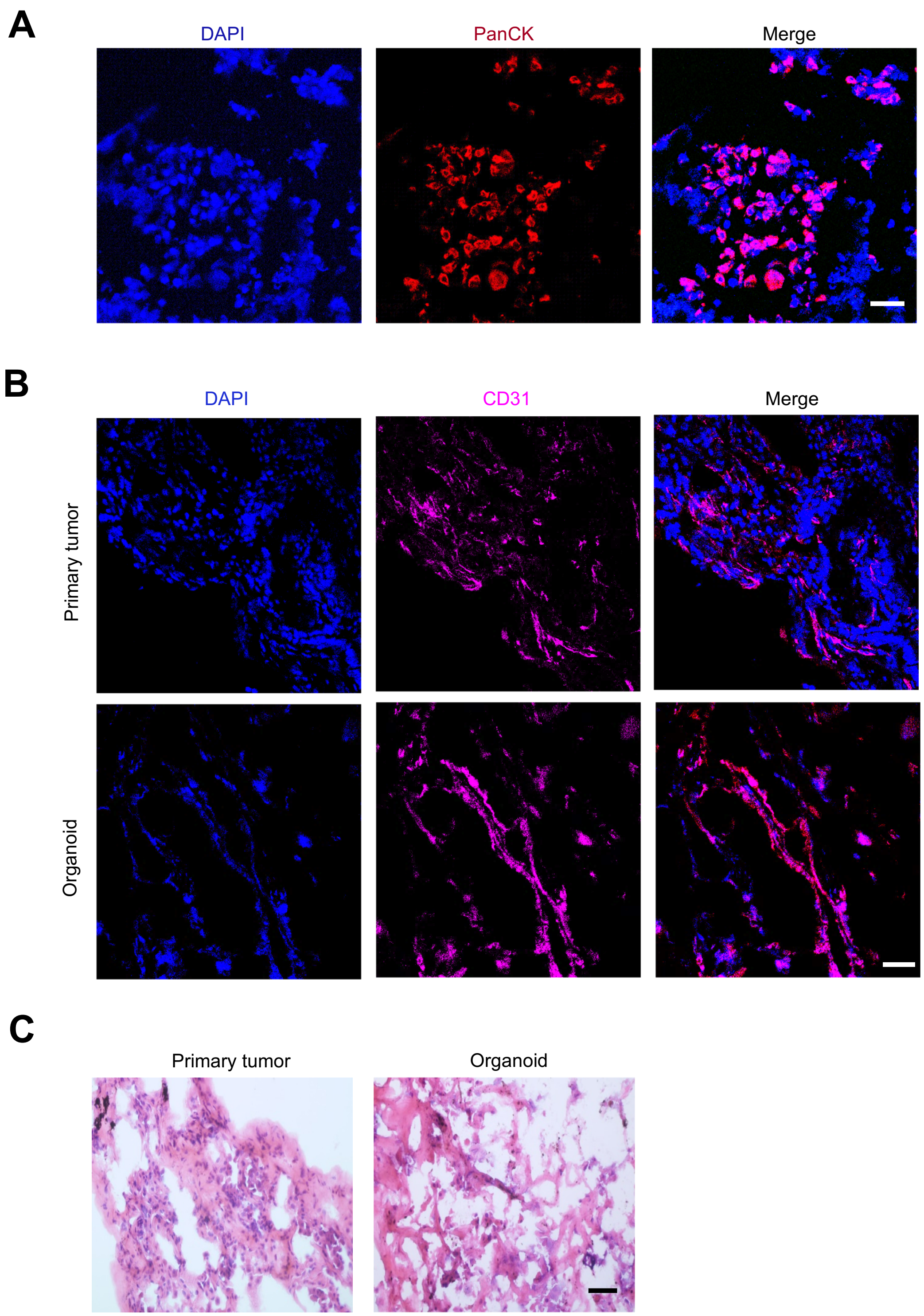

Fig. S7 Establishment of NSCLC patient-derived organoid (PDO)

(A) Representative of 5 PDOs with immunostaining for PanCk. Nuclei were stained with DAPI. Scale bar, 100 μm. (B) Representative of 5 PDO-primary tumor pairs with immunostaining for CD31. Nuclei were stained with DAPI. Scale bar, 100 μm. (C) Representative HE staining of 5 PDO-primary tumor pairs. Scale bars, 20 μm.

Supplementary Figure 8

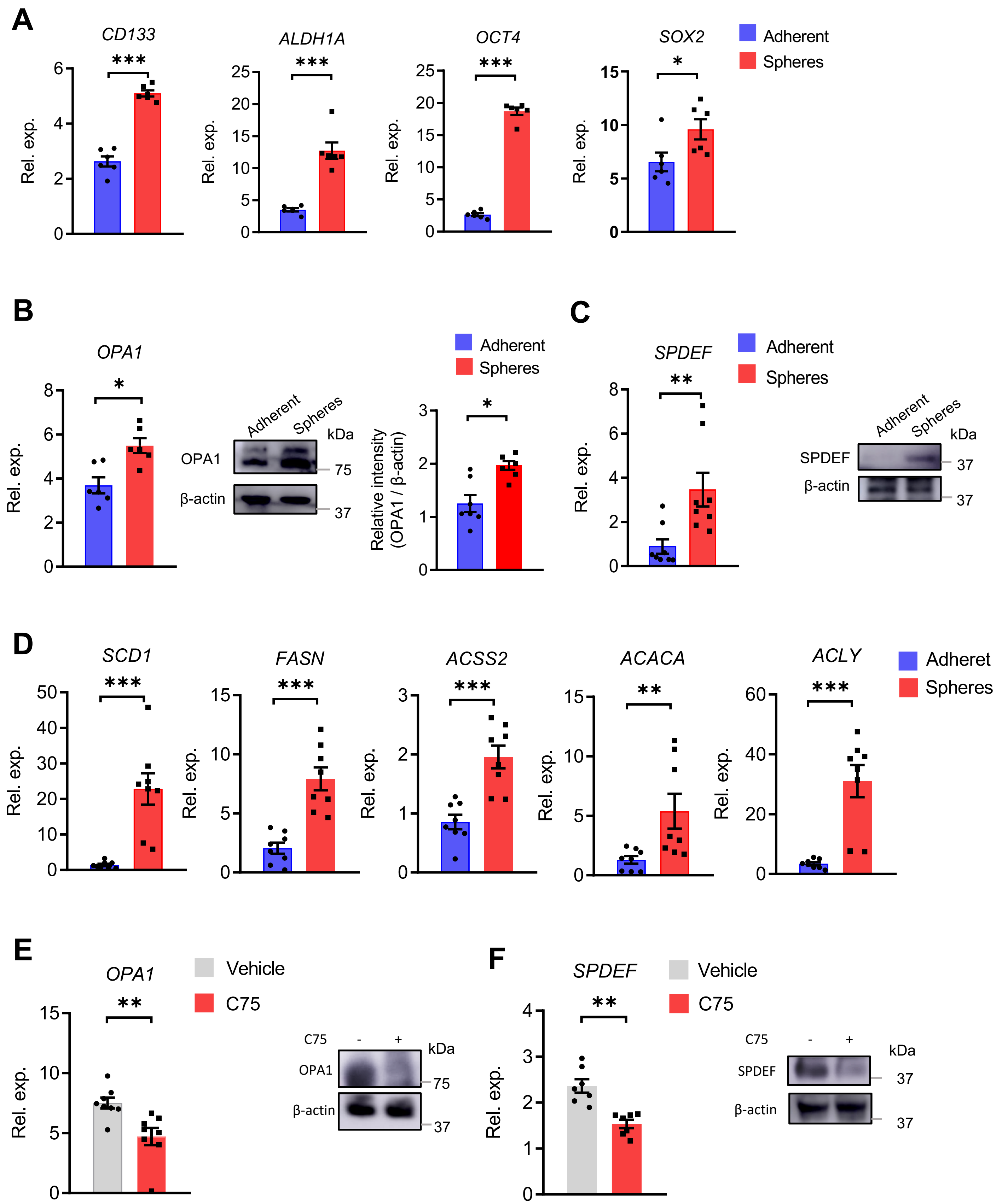

Supplementary Figure 8

Fig. S8 Lipogenesis licenses SPDEF^{high} and OPA1^{high} in Calu-1 stem-like cells

(A) Calu-1 tumor spheres were enriched with stem-like properties. Mean±SEM from 6 independent experiments. (B-C) Elevated mRNA and protein levels of OPA1 and SPDEF in Calu-1 tumor spheres. Representative and mean±SEM from 6-8 independent experiments. (D) Lipogenesis-related genes were upregulated in Calu-1 tumor spheres. Mean±SEM from 8 independent experiments. (E-F) C75 treatment reduced the expressions of OPA1 and SPDEF in Calu-1 tumor spheres. Representative and mean±SEM from 7-8 independent experiments. *$p < 0.05$, **$p < 0.01$ and ***$p < 0.0001$ with paired t-test.
