# Supplementary material for: Lipogenesis promotes mitochondrial fusion and maintains cancer stemness in human NSCLC
Source: JCI Insight. 2023 Mar 22;8(6):e158429. doi: 10.1172/jci.insight.158429 (PMC10070109; doi:10.1172/jci.insight.158429)
Supplement: Supplemental tables 1-4 [file jciinsight-8-158429-s144.pdf]

Supplementary Table 1

A list of elevated genes involved in lipid metabolism of tumor spheres

SYMBOL	baseMean_NON_CSC	baseMean_CSC	log2FoldChange	pval	padj
OLAH	0.306474062	72.79980383	7.892025775	2.60541E-15	1.92061E-14
THEM5	0.574009322	124.5464748	7.761394305	5.05036E-31	6.76707E-30
CD36	0.574009322	34.66130714	5.916108084	1.91545E-09	9.62168E-09
ACOX2	8.07712472	309.5807357	5.260330073	1.1809E-105	5.427E-104
ALOX5	0.880483384	31.0464566	5.139989031	3.1923E-12	1.95787E-11
CYP11A1	0.334187691	6.106590969	4.191636713	0.008109642	0.018468948
STARD4	265.6723168	4552.050095	4.098796623	3.95679E-76	1.27427E-74
ACBD7	13.49902443	182.3426411	3.755724924	2.05626E-49	4.37532E-48
CYP39A1	3.98118186	50.34053194	3.660451781	1.13452E-14	8.05025E-14
GDPD5	62.91162217	733.1396356	3.542689536	1.27653E-47	2.58436E-46
DHCR24	769.9713299	8932.929105	3.536256679	0	0
ACOT4	4.6855151	51.09925094	3.447022579	3.83277E-14	2.63881E-13
CYP4F11	218.0801835	2188.424773	3.326962206	1.9197E-155	1.5035E-153
SPNS2	43.80027875	422.5219895	3.27001447	6.50755E-62	1.69889E-60
MSMO1	639.7137837	6083.986707	3.249518527	0	0
LIPH	197.3460328	1586.288207	3.006855491	1.4149E-214	1.6278E-212
BCHE	69.83291528	532.7528644	2.931487338	6.45446E-94	2.5515E-92
PPARGC1A	13.20675649	98.04549851	2.892175203	8.91462E-16	6.71697E-15
ARSG	16.98500011	124.9598422	2.879131403	5.26541E-26	6.09984E-25
MVD	408.0654116	2558.952634	2.648681108	1.4742E-178	1.3293E-176
PLEKHA2	185.6789653	1134.553573	2.611242441	9.8235E-111	4.7777E-109
CDS1	23.82636021	134.4244309	2.496164881	2.34887E-18	1.98458E-17
IDI1	1021.030221	5667.904523	2.472789888	6.9008E-284	1.2247E-281
ACSBG1	1.493431508	8.217589372	2.460084168	0.021837494	0.045752225
FDFT1	1659.392677	9087.97834	2.453304072	0	0
LPIN1	447.0744201	2441.440208	2.449145538	3.5501E-201	3.7015E-199
SPTSSA	1255.462611	6814.86365	2.440465727	7.8416E-294	1.4698E-291
SQLE	1735.056178	9086.608412	2.388759531	9.2096E-303	1.8736E-300
SGPP2	52.20562927	269.9770312	2.370559388	1.03932E-08	4.87568E-08
SLC27A2	632.5097833	3172.801217	2.326597436	2.819E-138	1.8514E-136
ACOT11	22.45110191	110.9712697	2.305328053	5.29119E-09	2.54008E-08
HMGCR	1066.488624	5237.692113	2.296062679	6.9033E-246	9.6774E-244
ACSL5	4.00889549	19.33377878	2.269846927	0.000441933	0.001251243
SC5D	396.4332125	1829.571117	2.206355756	3.2052E-147	2.3147E-145
TM7SF2	80.45642549	350.084092	2.121421958	1.24787E-33	1.80546E-32
CYP1A1	2.708102583	11.72724134	2.114509387	0.011244635	0.024974438
TRIB3	800.2212904	3436.059332	2.10228403	4.18673E-82	1.46115E-80
FAAH	8.348997726	35.75431253	2.098442342	4.59596E-06	1.67937E-05
CYP1B1	344.4156546	1466.832945	2.090481951	2.1459E-08	9.81034E-08

PLBD1	5.471632424	23.19228067	2.083601477	0.000294664	0.00085742
GPCPD1	798.8128258	3383.710726	2.082676836	4.37414E-45	8.40661E-44
LSS	647.6003858	2736.012364	2.078899	1.52E-163	1.2807E-161
PLAAT4	8.986678535	37.2287932	2.05055895	4.19456E-06	1.54025E-05
HMGCS1	1303.616477	5346.148142	2.035980323	2.01181E-74	6.30847E-73
ABCD1	79.38585318	318.4717176	2.004211415	1.46878E-35	2.24997E-34
FDPS	515.599592	2050.605972	1.991727274	2.6423E-133	1.6571E-131
SCD	18191.00694	70076.17302	1.945698586	4.8006E-253	7.0871E-251
PNPLA3	62.23152734	237.6312635	1.933007095	2.77317E-27	3.34264E-26
DHCR7	1117.348832	4141.651342	1.890126448	1.7882E-163	1.499E-161
MVK	253.5102297	923.5721883	1.865180765	3.84735E-74	1.2019E-72
HSD17B7	179.3356284	644.13494	1.844700815	2.58452E-56	6.18578E-55
ACSL1	549.1618647	1947.989463	1.826682525	7.6323E-113	3.7894E-111
SREBF2	1101.471941	3897.505661	1.823118374	5.8289E-151	4.3604E-149
CYP4F3	357.6134934	1258.67018	1.815427213	1.69386E-86	6.18316E-85
FASN	6919.168651	24032.86936	1.796338296	7.8036E-208	8.6211E-206
ACAT2	451.6911643	1557.574822	1.78589287	3.37479E-96	1.36315E-94
MBOAT1	48.5559215	166.0437204	1.773844012	1.5331E-17	1.2543E-16
STS	103.6657042	353.2040321	1.768563128	2.7338E-33	3.89789E-32
PTGS2	882.1647972	2972.311704	1.752465322	1.76679E-80	6.00278E-79
PIK3R3	47.84036309	158.8360683	1.731238311	2.73823E-16	2.11772E-15
INSIG1	580.8722798	1868.658392	1.685709966	1.44596E-96	5.89767E-95
HELZ2	289.4650241	923.6670778	1.673983908	5.51711E-25	6.13576E-24
ACADL	5.613181514	17.88785486	1.67208977	0.006639314	0.015410746
GPD1	7.178528737	22.16729105	1.626672383	0.002881762	0.007143174
ACHE	14.71233871	45.32393689	1.623246582	2.18526E-05	7.39441E-05
FADS2	4293.969845	13061.70797	1.604959594	6.5469E-113	3.2602E-111
SLC44A5	26.64303497	78.46349301	1.558263129	5.47731E-07	2.1954E-06
ABHD3	361.7879091	1064.944043	1.557561527	3.37501E-61	8.74254E-60
PLA2G6	49.82416876	145.3400078	1.544514247	3.95402E-13	2.5596E-12
PLPP1	422.9861409	1232.516857	1.542925079	0.003025998	0.007470726
PISD	334.5046436	925.052059	1.46750832	8.40629E-51	1.83073E-49
AHR	394.3108992	1074.262082	1.445940509	5.941E-55	1.38033E-53
TXNRD1	25169.98102	68417.89292	1.442669545	6.4727E-125	3.8021E-123
PNPLA4	73.84777266	200.2834415	1.439416837	2.3082E-15	1.70832E-14
ACOT1	28.14726039	75.2627431	1.418941363	3.87514E-06	1.42894E-05
ELOVL6	685.505656	1779.889017	1.376546811	1.17273E-43	2.18587E-42
FAR2	23.26655701	59.65853068	1.35847071	1.99623E-05	6.7837E-05
ACSL4	2037.217992	5180.660168	1.346535588	3.17296E-96	1.28474E-94
HSD11B2	9.558405517	23.96930592	1.326346253	0.00903605	0.020422623
PCYT2	265.4718878	658.0229707	1.309578853	2.98772E-33	4.24903E-32
GM2A	625.2285183	1539.879669	1.300362129	3.77915E-56	8.9934E-55
FHL2	873.5234482	2146.870364	1.297316743	1.37291E-65	3.77313E-64

PNPLA7	42.62226414	102.7816488	1.269903564	1.14945E-05	4.02164E-05
PLA2G4A	924.2482078	2228.382685	1.269644765	2.33625E-50	5.03532E-49
NSDHL	686.8201051	1648.224303	1.262908412	3.44815E-55	8.05632E-54
AKR1B15	171.6992081	411.2626269	1.260176588	3.8374E-22	3.80365E-21
SMPD1	104.7386218	245.3927726	1.228299232	1.97986E-08	9.06863E-08
SUMF1	300.2599938	703.2304223	1.227785219	9.85311E-32	1.34509E-30
PLA2R1	251.8390643	575.7353707	1.192903762	2.06177E-21	1.99852E-20
PRKAA2	383.4043312	864.0054512	1.17217378	9.07126E-34	1.3251E-32
EPHX2	130.2528806	292.5743714	1.16748812	3.78412E-15	2.75904E-14
LPGAT1	3198.568944	7095.427598	1.149463049	2.38376E-39	4.00462E-38
HEXB	1319.144238	2889.643186	1.131289039	2.15512E-58	5.37395E-57
ASAH1	1172.589348	2566.097335	1.129878039	3.58247E-37	5.72576E-36
SELENOI	1546.753583	3384.245412	1.129590815	2.65724E-53	6.04749E-52
SAMD8	721.7795695	1559.725809	1.111662222	2.48089E-43	4.57812E-42
ESYT3	61.57920915	131.7389931	1.097167184	2.21388E-07	9.18706E-07
HACL1	260.9997745	556.4911092	1.092310078	2.82318E-22	2.81845E-21
AACS	623.4024828	1328.719214	1.091800459	1.93722E-38	3.19651E-37
ELOVL5	3108.163638	6526.963396	1.070349488	4.34697E-67	1.23748E-65
NEU1	255.4729822	527.3047178	1.045466179	4.32827E-15	3.14118E-14
CYP24A1	11616.65358	23750.33794	1.031753511	1.56588E-61	4.06252E-60

Supplementary Table 2

Demographic and clinical characteristics of NSCLC patients

	**Patients with NSCLC**
**Demographic parameters**	
No. of subjects	23
Sex (F/M)	19/4
Age (mean ± SEM [years])	60.17 ± 6.99
TNM staging (I/II/III)	9/6/8
Disease duration (mean ± SEM [months])	3.49 ± 3.59
**Histological types**	
Adenocarcinoma	15
Squamous carcinoma	8

Supplementary Table 3

Sequence of primers targeting different genes for qPCR

**Oligo name**	**Forward primer (5' to 3')**	**Reverse primer (5' to 3')**
18SrRNA	AGTCCCTGCCCTTTGTACACA	GATCCGAGGGCCTCACTAAAC
OPA1	TGTGAGGTCTGCCAGTCTTTA	TGTCCTTAATTGGGGTCGTTG
ACACA	AGTGGGTCACCCCATTGTT	TTCTAACAGGAGCTGGAGCC
SCD1	GCAGCCGAGCTTTGTAAGAG	GTTCTACACCTGGCTTTGGG
FASN	GAAGCTCGTGTTGACTTCTC	AGAAGACCACAAAGTAGTCC
ACSS1	ACCAAGATCGCCAAATATGC	TGCTTGTCCTTGCACTTCTG
ACSS2	GGATTCCAGCTGCAGTCTTC	CATGCCACCACAAGTCAATC
ACLY	GGTGCTCCGGATTTTGC	ACATGGCTGCAGAGAGACCT
SPDEF	TGTCCGCCTTCTACCTCTCCTAC	CGATGTCCTTGAGCACTTCGC
Jun	CCTTGAAAGCTCAGAACTCGGAG	TGCTGCGTTAGCATGAGTTGGC
TEAD4	GAAGGTCTGCTCTTTCGGCAAG	GAGGTGCTTGAGCTTGTGGATG
MAFK	CTGCGCTCCAAGTACGAGGCG	TCGGTGGACTTGACGATGGTGA
CEBPBeta	AGAAGACCGTGGACAAGCACAG	CTCCAGGACCTTGTGCTGCGT
ELF1	CTAAAGCAGTGTCCAGGTTGTGG	CGCTGACCTTCCACTTTTGCCA
CD133	GCACTCTATACCAAAGCGTCAA	CTCCCATACTTCTTAGTTTCCTCA
ALDH1A1	CGCAAGACAGGCTTTTCAGAT	CCCTCTCGGAAGCATCCA
OCT4	ACATCAAAGCTCTGCAGAAAGAAC	CTGAATACCTTCCCAATAGAACC
SOX2	TACAGCATGTCCTACTCGCAG	GAGGAAGAGGTAACCACAGGG
HSD17B4	GAAGTCCCCTCCCAAATCAT	GTGGTACTGGTCACCGGC
ECHS1	TTTTCTGCGATGATGTACTCAAA	CTGCGTGTCCTGCTGTCCT
EHHADH	CTGAATTGGCTTGTTGCAGA	CTCAGACCCGGTTGAAGAAG
ACADS	CCCATCTTCTTCACCTGAGC	AGATGTTGCTCCAGACATGC
ACAA1	CACTCAGAAACTGGGCGATT	CTCAAGGACGTGAATCTGAGG
CPT1A	GCCTCGTATGTGAGGCAAAA	TCATCAAGAAATGTCGCACG
ACADL	TCATGCAGCTGGAGACAGTT	TTGGCAAAACAGTTGCTCAC
HADHSC	ACTGTGTGACCAGTTGCTGC	GCCTCGGCCAAGAAGATAAT
ACACB	CAGCTTCTTGTGTTCCCGTC	CCTGGAGGCTTATCTGACCA
HADHA	CCGTTCCTCTGGAGGTTTTA	TGGTAGAAGCATTCGTGCAG
CPT2	CGGAGTCTCGAGCAGATAGG	GGAAAAGAACTGCATGAGCA

Supplementary Table 4

Sequence of primers targeting OPA1 promoter regions in ChIP-PCR

Oligo name	Forward primer (5' to 3')	Reverse primer (5' to 3')
OPA1	TTTATGGTGTTACCTTCCGTGA	AACTGGGACATAGCCCTAAATG
